# Entropy‐Driven Spin Transition in Rare Earth Perovskites Enables Feedback Adsorption for Enhanced Acidic Water Oxidation

**DOI:** 10.1002/adma.73021

**Published:** 2026-04-08

**Authors:** Yong Jiang, Mingzi Sun, Zhong Liang, Hao Fu, Ziyun Zhong, Bolong Huang, Yaping Du

**Affiliations:** ^1^ Tianjin Key Lab for Rare Earth Materials and Applications Center for Rare Earth and Inorganic Functional Materials School of Materials Science and Engineering National Institute for Advanced Materials Nankai University Tianjin China; ^2^ Department of Chemistry City University of Hong Kong Kowloon Hong Kong SAR China

**Keywords:** high entropy, lattice oxygen mechanism, rare earth, spin regulation, water oxidation

## Abstract

Modulating catalytic reaction pathways and site reaction behaviors to break the activity/stability trade‐off poses significant challenges for the acid oxygen evolution reaction (OER). Herein, a sol‐gel method is proposed to prepare high entropy rare earth (HERE) perovskite oxides HERECoO_3_/RuO_2_ (RE = La, Pr, Nd, Sm, Eu, Gd, Tb, Dy, Ho, Er, Tm, Yb, and Lu) for pH‐universal OER for the first time. (LaPrNdSmEu)CoO_3_/RuO_2_ achieves a current density of 10 mA cm^−2^ for OER with overpotentials of only 115 mV and operates stably over 1000 h at 0.1 A cm^−2^ under the acidic condition. Experimental results indicate that the novel spin regulation‐lattice oxygen mechanism (SR‐LOM) induces a shift in the OER mechanism from the adsorption evolution mechanism (AEM) to LOM, and promotes the spin state transition of Co to optimize intermediate adsorption. Theoretical calculations have confirmed that the high entropy strategy has induced stronger interactions at the heterointerface, which not only accelerates the electron transfer but also promotes the electroactivity of the surface. Moreover, the lattice oxygen becomes more flexible in HERECO_3_/RuO_2_, enabling the LOM process to promote the superior OER with reduced energy barriers. Our findings provide a new way for the rational design of highly active RE‐based electrocatalysts.

## Graphical abstract

A synthetic protocol for fabricating high‐entropy rare earth perovskite oxides with abundant lattice distortions and oxygen vacancies has been developed, representing an emerging platform for anchoring RuO_2_. High entropy strategy significantly modulated the electronic structures and spin states, inducing a shift in the mechanism from the adsorption evolution mechanism to the lattice oxygen mechanism, achieving remarkable oxygen evolution reaction performance.

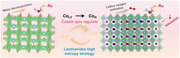

## 1 Introduction

Compared with traditional alkaline water electrolysis, proton exchange membrane (PEM) water electrolysis offers advantages such as higher current density, higher hydrogen purity, and relatively lower energy consumption, making it an ideal technology for large‐scale hydrogen production from renewable energy in the future. In a PEM electrolyzer, water undergoes an oxidation reaction at the anode (i.e., acidic oxygen evolution reaction, OER), producing oxygen, protons, and electrons. Therefore, the acidic OER serves as the cornerstone of PEM water electrolysis technology [1, 2, 3, 4]. However, the shortcomings of slow kinetics and low overpotential to achieve the high current density of the OER process limit the scale‐up of water splitting [5, 6, 7, 8, 9, 10, 11]. However, despite significant efforts made by researchers to accelerate the multi‐electron reaction process of electrode surface hydrolysis and proton generation, there are still some issues that have not received sufficient attention, such as changes in ion concentration and slow kinetics during the reaction process. Therefore, the development of high‐performance electrocatalysts with high efficiency, low cost, and stability is still facing serious challenges. Currently, precious metal catalysts (Pt and Pt‐based catalysts) [12, 13, 14]. are used to achieve high‐performance hydrogen evolution reaction (HER), but the high cost of use and unsatisfactory OER activity limit their wide application [15, 16]. On the other hand, iridium (Ir)/ruthenium (Ru) oxide is generally considered to be an excellent OER electrocatalyst, but its stability and HER performance are poor. Based on the above considerations, the development of new electrocatalysts that can strike a good balance between reactivity, cost, and stability has become the focus of extensive research in the field of electrocatalysis.

So far, the catalytic mechanisms of the OER reaction are mainly divided into two types, including the traditional adsorbent evolution mechanism (AEM) and the dynamic favorable lattice oxygen mechanism (LOM) [17, 18, 19]. In general, two reaction mechanisms, AEM and LOM, often coexist and compete during the OER process. Due to the direct combination of lattice oxygen with oxygen‐containing intermediates, the theoretical voltage of the LOM is lower than that of AEM, which breaks the limit of the inherent potential of AEM [20, 21], therefore, catalysts with LOM generally have superior catalytic activity than AEM. In addition, unlike the stable active center in AEM, the catalyst with LOM usually has no defined active site, and its structure is always in dynamic change during the OER. Yan et al. studied the reaction mechanism of La_x_Sr_1‐x_CoO_3‐x_ with different structures by using OER as a model [22]. The oxygen vacancy content of the catalyst is regulated by precisely controlling the doping amount of Sr in the catalyst, so that the OER mechanism of La_x_Sr_1‐x_CoO_3‐x_ can be converted between AEM‐LOM‐AEM. Although LOM has a major contribution to catalyst activity, it also seriously affects the electrochemical stability of catalysts [23, 24]. Although the formation of oxygen vacancies and accelerates the dissolution of metals, thus triggering the collapse of the catalyst structure, but also results in rapid deactivation of the catalyst [25]. High‐entropy materials are composed of multiple elements (typically five or more) in nearly equal proportions. The high‐entropy effect promotes the formation of simple and uniform solid solution phases within the catalyst, avoiding compositional segregation and phase separation, thus imparting enhanced reaction stability [26, 27]. Therefore, incorporating high‐entropy strategies into the perovskite rare earth (RE) site structure not only facilitates the maintenance of long‐term stability of the catalyst but also avoids shadowing the clear elucidation of the reaction behavior at the active sites.

Additionally, RE elements with unique 4f orbitals are commonly considered as dopants to enhance the performance of perovskite due to their chemical properties and atomic size similar to La [28, 29]. Gao et al. induced the transition of the Co 3p spin state from the low spin state (LS) to the intermediate spin state (IS) by doping Ce in the La position in LaCoO_3_, resulting in enhanced covalency of Co 3d‐O 2p and improved conductivity [30]. Shao‐Horn et al. proposed that the IS Co^3+^ (e_g_ = 1) can optimize the binding of oxygen‐containing related intermediate groups on octahedral cations, thereby improving the intrinsic OER performance [31]. The aforementioned research results indicate that doping at the RE site of LaCoO_3_ can alter the reaction mechanism (from AEM to LOM), effectively enhancing the catalyst's activity, or change the spin state of the octahedral central Co to promote the adsorption of oxygen‐containing intermediates. However, it has been rarely reported in direct studies on the effect of spin state transitions on OER performance and stability within the LOM mechanism.

Herein, a series of high entropy RE (HERE) perovskite oxides HERECoO_3_/RuO_2_ (RE = La, Pr, Nd, Sm, Eu, Gd, Tb, Dy, Ho, Er, Tm, Yb, and Lu) were prepared for the first time by a facile sol‐gel assisted one‐pot solvent reduction method, and used as pH‐universal water oxide catalyst. Based on the existing experimental and characterization results, a novel spin regulation‐lattice oxygen mechanism (SR‐LOM) was proposed to explain the excellent OER performance and stability. Taking (LaPrNdSmEu)CoO_3_/RuO_2_ as an example, the high entropy strategy of RE elements in the La site changes the reaction mechanism of the catalyst (AEM to LOM) and improves the catalytic activity. Importantly, Abberation‐Corrected Transimission Electron Microscopy (AC‐TEM) results show that the severe lattice distortion induced by high entropy induces the spin state of the central Co of the octahedron to shift from LS to IS, which enhances the adsorption of oxygen‐containing intermediates and enhances the reaction stability of the catalyst. In addition, the introduction of the Ru site promoted the dissociation of water and increased the concentration of oxygen‐containing species. Thanks to a special SR‐LOM reaction mechanism, the (LaPrNdSmEu)CoO_3_/RuO_2_ can deliver a current density of 10 mA cm^−2^ with an overpotential of 115 mV in 0.5 M H_2_SO_4_ solutions. Additionally, benefits from the special structure, the prepared catalyst exhibits excellent activity and stability, achieving a current density of 400 mA cm^−2^ at only 163 mV in 0.5 M H_2_SO_4_, and can operate stably for 1000 h at a current density of 0.1 A cm^−2^. Density functional theory (DFT) calculations demonstrate that the introduction of high‐entropy RE elements in La sites has significantly modulated the electronic structures and spin states of metal sites. Accordingly, the electron transfer is promoted through the interfaces by the RE sites, and the lattice oxygen also becomes more electroactive to initiate the LOM for OER toward remarkable performances.

## 2 Results and Discussions

**FIGURE 1:**
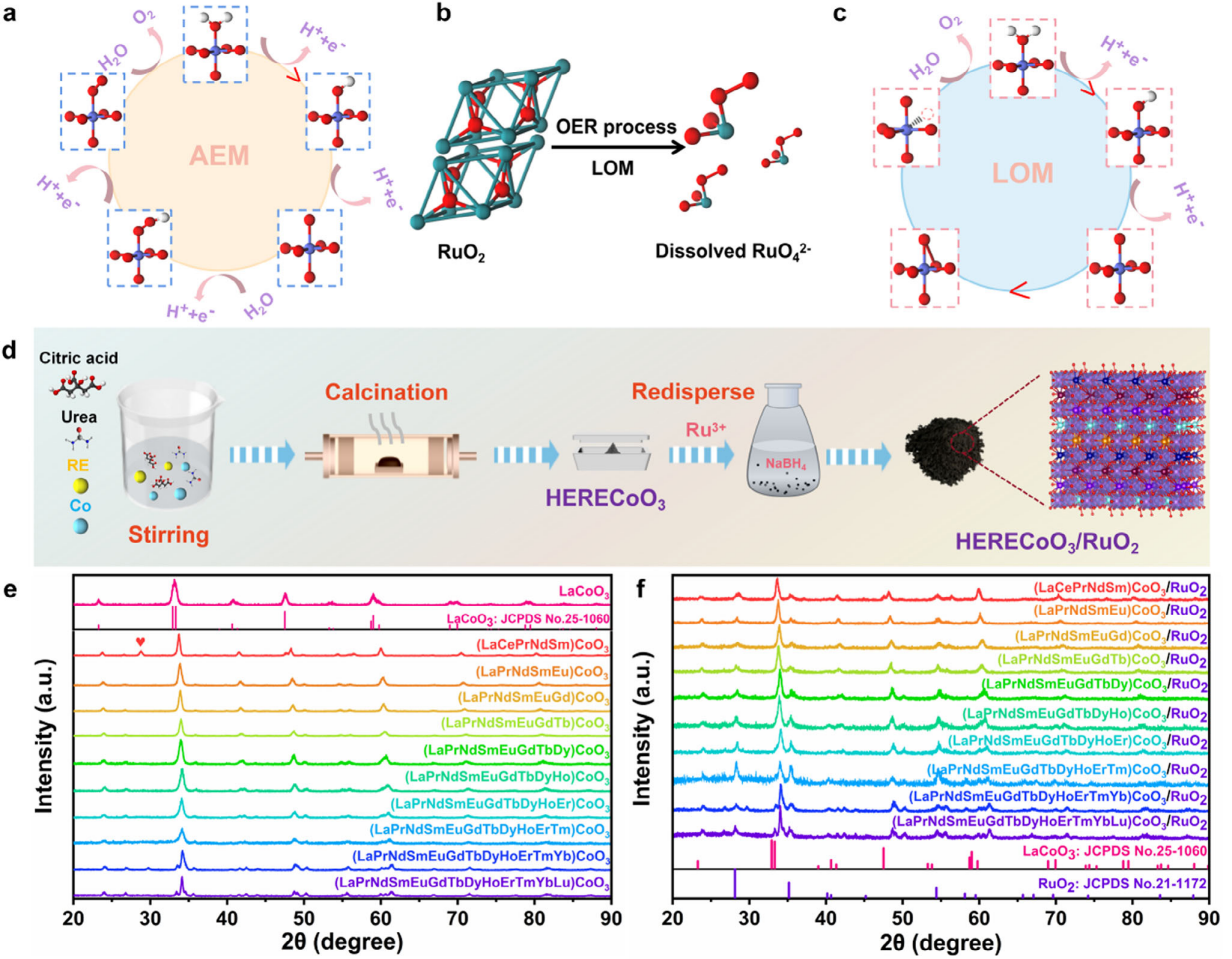
(a–c) The illustration of AEM, transformation of RuO_2_ to RuO_4_^2^^−^ and LOM of catalyst toward the OER. (d) Schematic diagram of preparation process of (HEREs)CoO_3_/RuO_2_. XRD patterns of (e) (HEREs)CoO_3_ and (f) (HEREs)CoO_3_/RuO_2_.

Figure 1a shows the typical AEM pathway, where water dissociates at the metal active site and then evolves to form oxygen molecular spillover by adsorption. As a widely recognized benchmark catalyst for OER, RuO_2_ exhibits excellent electrochemical activity. However, it suffers from severely compromised stability due to the formation of high‐valence Ru oxides under high‐potential conditions during the LOM pathway, ultimately leading to structural degradation (Figure 1b). The LOM pathway usually involves direct O─O coupling between the catalyst and lattice oxygen, resulting in lattice oxygen participation, which is more favorable in OER kinetics (Figure 1c). By means of rational and precise structural design of the OER catalyst, it is possible to simultaneously achieve outstanding catalytic activity and reaction stability. Herein, the precursor of HERE perovskite oxide was prepared by a simple sol‐gel method, and then (LaPrNdSmEu)CoO_3_ was obtained after being treated in the air at 600°C for 6 h. RuCl_3_ solution was ultrasonically mixed with the obtained HERE perovskite oxide, and then NaBH_4_ was slowly dropped into the mixture. After stirring, filtering, drying, and then annealing at 600°C in an air atmosphere for 3 h to obtain (LaPrNdSmEu)CoO_3_/RuO_2_ (Figure 1d). Powder X‐ray diffraction (XRD) was performed to obtain the structural information of LaCoO_3_. As shown in Figure S1, the XRD patterns of catalysts treated under different calcination conditions indicate that calcination at either lower (400°C) or higher temperatures (1000°C) results in amorphous and highly crystalline structures (with lower oxygen vacancy concentrations), which increases the complexity of regulating catalytic performance [32]. Moreover, the characteristic diffraction peaks of both LaCoO_3_ and RuO_2_ can be found in the spectra of LaCoO_3_/RuO_2_, which also indicates the successful synthesis of LaCoO_3_/RuO_2_ heterostructures (Figure S2a) [33, 34, 35]. Moreover, the heterogeneous structures of LaCoO_3_ and RuO_2_ with different ratios were also considered and successfully synthesized, which was further confirmed by XRD results (Figure S2b). The transmission electron microscopy (TEM) images confirmed that the structures constructed with different ratios of LaCoO_3_ and RuO_2_ were still well maintained (Figure S3). The scanning electron microscopy (SEM) images were also used to verify the microstructure and corresponding element distribution of La, Co, O, and Ru over LaCoO_3_ and LaCoO_3_/RuO_2_ (Figures S4 and S5). Additionally, a series of HERE perovskite oxides were also prepared by the same synthesis method as LaCoO_3_, and the XRD results show that there was no significant difference in the structure of the catalysts with different amounts of RE elements (HERECoO_3_), which also demonstrated the universality of this synthetic protocol (Figure 1e; Figure S6) [36, 37]. It should be noted that a characteristic diffraction peak located at 28.5° belonging to CeO_2_ was observed in the XRD pattern of (LaCeNdSmEu)CoO_3_, which indicates that the introduction of the Ce element breaks the stable high entropy structure. Furthermore, Ru^3+^ ions were impregnated on the surface of a series of HERECoO_3_ to obtain a series of precursors denoted as (HEREs)CoO_3_‐Ru, and both XRD patterns and TEM images revealed the uniform dispersion of Ru and the consistent morphology (Figures S7 and S8). The diffraction peak of RuO_2_ can also be clearly observed after calcination in an air atmosphere at 600°C, which further confirms the successful preparation of the heterostructure between RuO_2_ and HERECoO_3_ (Figure 1f). The observation of no significant difference in morphology as the number of RE elements at the La site in LaCoO_3_/RuO_2_ increases from five to thirteen confirms that the high‐entropy effect maintains its structural stability (Figures S9 and S10). Furthermore, inductively coupled plasma‐optical emission spectrometer (ICP‐OES) results determined that the atomic ratio of Ru was nearly equal (Table S1).

**FIGURE 2:**
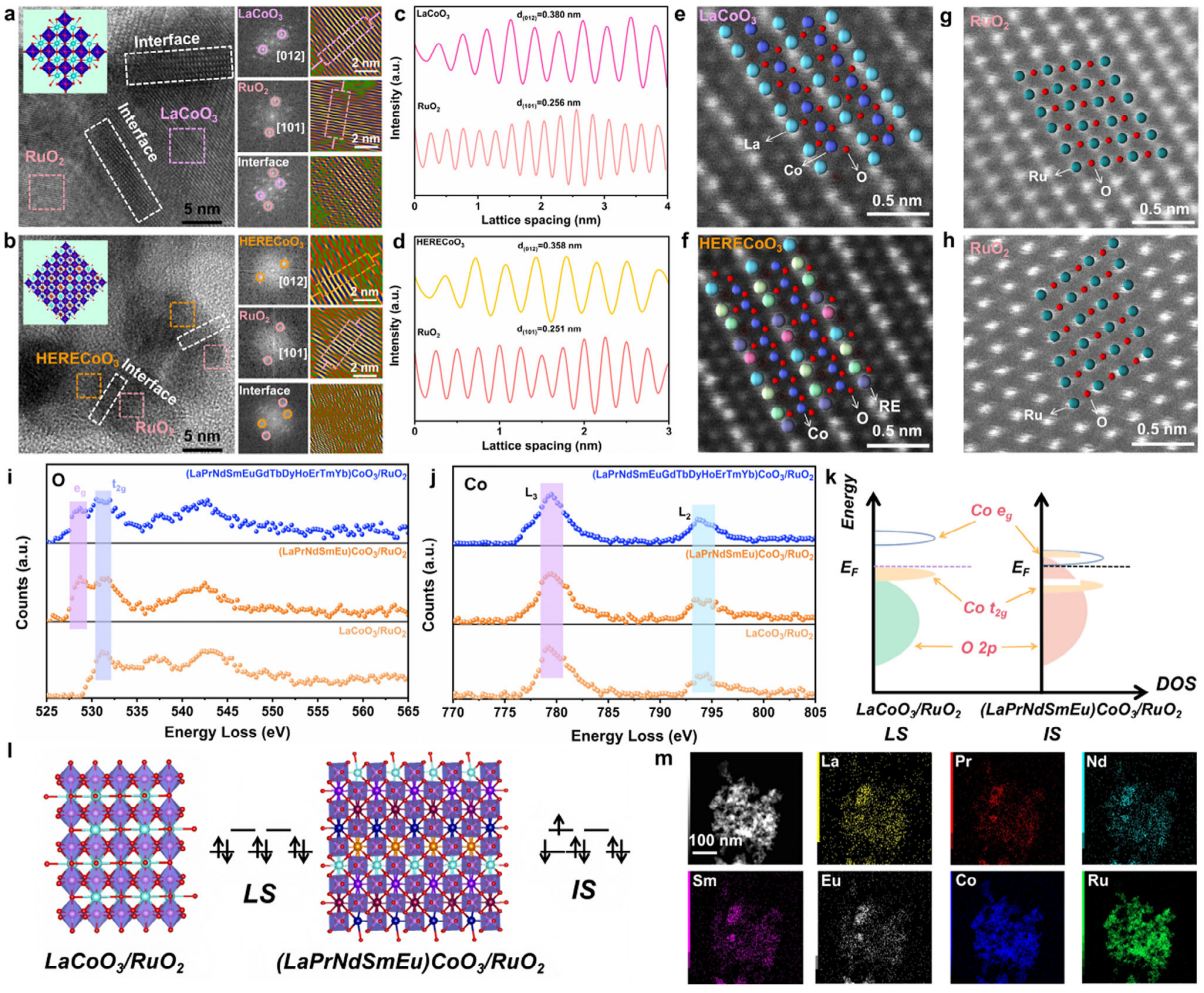
HR‐TEM images of (a) LaCoO_3_/RuO_2_ and (b) (LaPrNdSmEu)CoO_3_/RuO_2_. The intensity profiles (c) and (d) from the dashed areas in (a) and (b). (e–h) AC‐TEM images of LaCoO_3_/RuO_2_ and (LaPrNdSmEu)CoO_3_/RuO_2_ (the red balls represent O, the light purple balls represent Co, the cyan balls represent Ru, and the other colored balls represent RE). EELS analyses of (i) O K‐edge and (j) Co L‐edge for LaCoO_3_/RuO_2_, (LaPrNdSmEu)CoO_3_/RuO_2,_ and (LaPrNdSmEuGdTbDyHoErTmYb)CoO_3_/RuO_2_. (k) Schematic representation of Co 3d‐O 2p overlap for LaCoO_3_/RuO_2_ and (LaPrNdSmEu)CoO_3_/RuO_2_. (l) Schematic representation of the orbital splitting of Co 3d in for LaCoO_3_/RuO_2_ and (LaPrNdSmEu)CoO_3_/RuO_2_. (m) Elemental mapping for (LaPrNdSmEu)CoO_3_/RuO_2_.

Figure 2a,b of high‐resolution (HR)‐TEM images of LaCoO_3_/RuO_2_ display a clear lattice spacing of 0.380 and 0.256 nm, which corresponds to the (012) and (101) faces of LaCoO_3_ and RuO_2,_ respectively [30, 35]. It is worth noting that the hybrid interface of LaCoO_3_ and RuO_2_ can also be clearly observed on the HR‐TEM image, which is also consistent with the results of XRD. While the lattice spacing of (LaPrNdSmEu)CoO_3_/RuO_2_ is 0.352 nm, which may be due to lattice strain caused by the high entropy of RE elements at La positions in LaCoO_3_, the lattice shrinks from 0.380 to 0.352 nm (Figure 2c,d) [38, 39, 40, 41]. Moreover, the Aberration Corrected Transmission Electron Microscope (AC‐TEM) was used to further reveal the structure of LaCoO_3_/RuO_2_ and (LaPrNdSmEu)CoO_3_/RuO_2_ at the atomic scale (Figure 2e–h). Due to the serious lattice distortion of high‐entropy oxides, the atomic arrangement in (LaPrNdSmEu)CoO_3_/RuO_2_ is not as regular as that of LaCoO_3_, but the existence of lattice oxygen defects can still be observed, which is consistent with the previous characterization results. Furthermore, the electron energy loss spectroscopy (EELS) measurements are also used to detect the spin states of Co in different catalysts by measuring the O and Co L edges. The O─K edge intensity of (LaPrNdSmEu) CoO_3_/RuO_2_ is significantly lower than that of pure LaCoO_3_, due to the spin state of Co 3p from LS to IS (Figure 2i), causing electrons on the t_2g_ orbital to transition to the e_g_ orbital [42, 43]. Figure 2j shows the Co L_3_ and L_2_ edge peaks of LaCoO_3_, (LaPrNdSmEu)CoO_3_/RuO_2_ and (LaPrNdSmEuGdTbDyHoErTmYb)CoO_3_/RuO_2_ [44]. Importantly, the intensity of the Co L_3_ marginal peak of (LaPrNdSmEu)CoO_3_/RuO_2_ is lower than that of LaCoO_3_, which indicates that part of the e_g_ orbital of Co 3p in (LaPrNdSmEu)CoO_3_/RuO_2_ is filled to IS, which is also consistent with the results of Figure 2j [45]. The results show that the high‐entropy strategy of La site in LaCoO_3_ can induce the spin state transition of Co in LaCoO_3_, thereby improving its electrocatalytic activity. Further orbital analysis results, as shown in Figure 2k, indicate that the increased orbital overlap and occupation states near the Fermi level are due to the hybridization of the Co 3d and O 2p orbitals in (LaPrNdSmEu)CoO_3_/RuO_2_, resulting in an induced spin state transition of Co 3p from LS to IS. Based on the above considerations, the electron configuration diagram of Co in LaCoO_3_ and (LaPrNdSmEu)CoO_3_/RuO_2_ with IS was shown in Figure 2l, that is, the transformation of Co 3p from LS (e_g_ = 0) to IS (e_g_ = 1) promotes the adsorption of oxygen‐containing adsorbents by the octahedral Co center. In addition, energy dispersive spectroscopy (EDS) mapping showed that the element distribution in (LaPrNdSmEu)CoO_3_/RuO_2_ is uniform, once again indicating the successful preparation of (LaPrNdSmEu)CoO_3_/RuO_2_ (Figure 2m).

**FIGURE 3:**
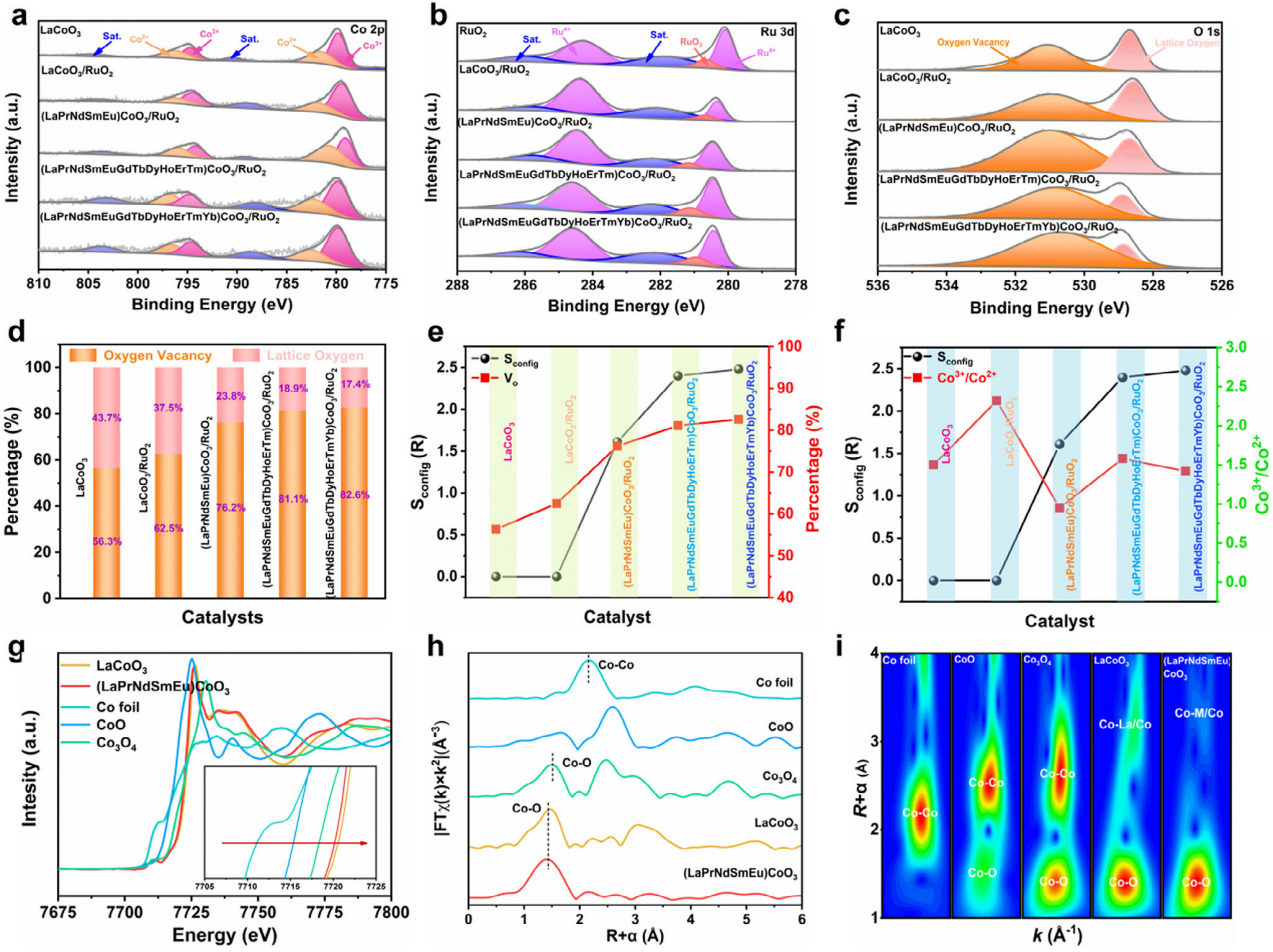
The XPS spectra for (a) Co 2p, (b) Ru 3d, and (c) O 1s of LaCoO_3_, LaCoO_3_/RuO_2_, (LaPrNdSmEu)CoO_3_/RuO_2_, (LaPrNdSmEuGdTbDyHoErTm)CoO_3_/RuO_2_ and (LaPrNdSmEuGdTbDyHoErTmYb)CoO_3_/RuO_2_. (d) Concentration of various O species. (e) The dependence of S_config_ and the V_O_ concentration in various catalysts, and (f) the dependence of S_config_ and the Co^3+^/Co^2+^ ratio in various catalysts. (g) Co K‐edge XANES and the fitted average oxidation states of Co in LaCoO_3_/RuO_2_, (LaPrNdSmEu)CoO_3_/RuO_2_ with Co‐foil, CoO, and Co_3_O_4_ as references. (h) Corresponding R space plots (i) Co K‐edge WT‐EXAFS contour plots of LaCoO_3_/RuO_2_, (LaPrNdSmEu)CoO_3_/RuO_2_, Co‐foil, CoO, and Co_3_O_4_.

The surface electronic structure and chemical state of the prepared samples were studied by X‐ray photoelectron spectroscopy (XPS). XPS spectrum of Co 2p, Ru 3d and O 1s in RuO_2_, LaCoO_3_, LaCoO_3_/RuO_2_, (LaPrNdSmEu)CoO_3_/RuO_2_, (LaPrNdSmEuGdTbDyHoErTm)CoO_3_/RuO_2_ and (LaPrNdSmEuGdTbDyHoErTmYb) CoO_3_/RuO_2_ were displayed in Figure 3a–c. The results indicate that after the formation of a heterogeneous interface between LaCoO_3_ and RuO_2_, there is a significant and strong electronic coupling, which is consistent with the XRD and HR‐TEM results. Compared to the binding energy positions of RuO_2_ to LaCoO_3_, the binding energies of Co and La show a negative shift, while that of Ru exhibits a positive shift, indicating electron transfer from RuO_2_ to LaCoO_3_ (Figure S11). Notably, as the number of RE elements substituting the La site in LaCoO_3_ increases, the direction of binding energy shifts for La, Co, and Ru in the catalyst changes. However, with the incorporation of more elements, the electron transfer behavior of the catalyst tends to stabilize, indicating that the high‐entropy effect is primarily confined to the optimization of the catalyst's electronic structure. (Figure S12). According to the AC‐TEM) results, severe distortion leads to a high concentration of lattice oxygen defects in the catalyst, making it essential to perform a detailed analysis of the O 1s fine spectrum of the sample. As shown in Figure 3d, with the increase of the entropy of the catalyst system, the oxygen vacancy concentration of the prepared sample increased from 62.5% to 82.6%, and the lattice oxygen vacancy concentration increased from 37.5% to 17.4%. Moreover, the lattice oxygen vacancy concentration tended to be saturated with the increase of entropy to the maximum, which also confirmed that the high entropy strategy endowed a regulation effect on the lattice oxygen defect concentration of the catalyst [46, 47]. Moreover, the electron paramagnetic resonance (EPR) spectra of the aforementioned samples indicate that the intensity of the oxygen vacancy signal (g = 2.003) increases progressively. This trend is consistent with the characterization results obtained from XPS, further confirming that the high‐entropy strategy effectively modulates the oxygen vacancy concentration in the catalysts. These findings establish a solid foundation for elucidating the crucial structure‐performance relationship of the catalysts (Figure S13). In addition, it can be seen from Figure 3e that considering the composition of La‐site and Co‐site and stoichiometry of O, the configuration entropy (S_config_) of the system also increases linearly from 1.61 to 2.56 R with the increase of the number of elements at the catalyst La site (5 to 13), which further indicates that the increase of RE elements can effectively regulate the configurational entropy of the catalyst. The dependence of S_config_ and the V_O_ concentration in LaCoO_3_, LaCoO_3_/RuO_2_, (LaPrNdSmEu)CoO_3_/RuO_2_, (LaPrNdSmEuGdTbDyHoErTm)CoO_3_/RuO_2_ and (LaPrNdSmEuGdTbDyHoErTmYb) CoO_3_/RuO_2_ indicate that the configurational entropy of the catalyst shows a significant positive correlation with the lattice oxygen defect, and with the entropy increases from 0 to 1.61 R, the concentration of the oxygen defect changes the most and then tends to be stable, which also confirms that the degree of distortion of the catalyst structure is the main factor limiting the lattice oxygen vacancy (Figure 3f). Additionally, the change relationship between S_config_, V_O,_ and Co^3+^/Co^2+^ also verified that the adoption of the high entropy strategy can further control the lattice oxygen defect concentration by regulating the structure of the catalyst, and promote the catalytic reaction performance. Furthermore, a comparison of the core‐electron ionization edges in the EELS results of Ru in LaCoO_3_/RuO_2_ and (LaPrNdSmEu)CoO_3_/RuO_2_ reveals that a higher oxygen vacancy concentration induces a shift of the Ru ionization edge (e.g., the L‐edge of transition metals) toward higher energy. This shift, accompanied by an increase in the Ru oxidation state (electron loss), leads to enhanced core‐electron binding energy. These results confirm that additional oxygen vacancies promote the elevation of the Ru oxidation state, thereby facilitating the OER process (Figure S14). A comparative study of the precise oxidation states and local atomic structures of LaCoO_3_ and (LaPrNdSmEu)CoO_3_ samples was conducted using X‐ray absorption spectroscopy (XAS). The Co K‐edge X‐ray absorption near edge structure (XANES) spectrum (Figure 3g) and the corresponding magnified white‐edge position indicate that the average valence state of Co in LaCoO_3_ is higher than that in (LaPrNdSmEu)CoO_3_, as well as in the reference samples of Co foil, CoO, and Co_3_O_4_. Additionally, LaCoO_3_ exhibits a lower content of high‐spin Co^2+^ compared to (LaPrNdSmEu)CoO_3_, which aligns with the results obtained from AC‐TEM and XPS analyses. The bonding environments of Co atoms were further investigated through Fourier‐transformed (FT) k^3^‐weighted χ(k) function (where k is wave number) and corresponding least‐squares data fitting (Figures S15 and S16 and Table S2). The Co k‐edge FT‐extended X‐ray absorption fine structure (FT‐EXAFS) spectrum (Figure 3h) exhibits two peaks at 1.92 and 3.32 Å, which can be assigned to the first coordination shell of Co─O and the second coordination shell of Co‐La/RE, respectively. Furthermore, the first coordination number (CN) around Co─O in LaCoO_3_ increases from 5.2 to 5.6 compared to that of (LaPrNdSmEu)CoO_3_. Additionally, the peaks mentioned above were further assigned using Cu K‐edge Wavelet Transform (WT)‐EXAFS spectrum (Figure 3i), indicating that the regions of (1.92 Å, 5.08 Å^−1^) and (3.32 Å, 7.3 Å^−1^) can be attributed to the first coordination shell of Co─O and the second coordination shell of Co‐La/RE in (LaPrNdSmEu)CoO_3_, respectively. The above results indicate that the increase in Co─O bond length (1.92 Å) and the decrease in Co valence state in (LaPrNdSmEu)CoO_3_ can elevate the Co spin state compared to LaCoO_3_ (1.90 Å). These spectroscopic results demonstrate that, with increasing configurational entropy, the octahedral Co centers become coordinated with various RE atoms, which induces a transition of the spin state from LS to IS, thereby facilitating the adsorption of catalytic intermediates.

**FIGURE 4:**
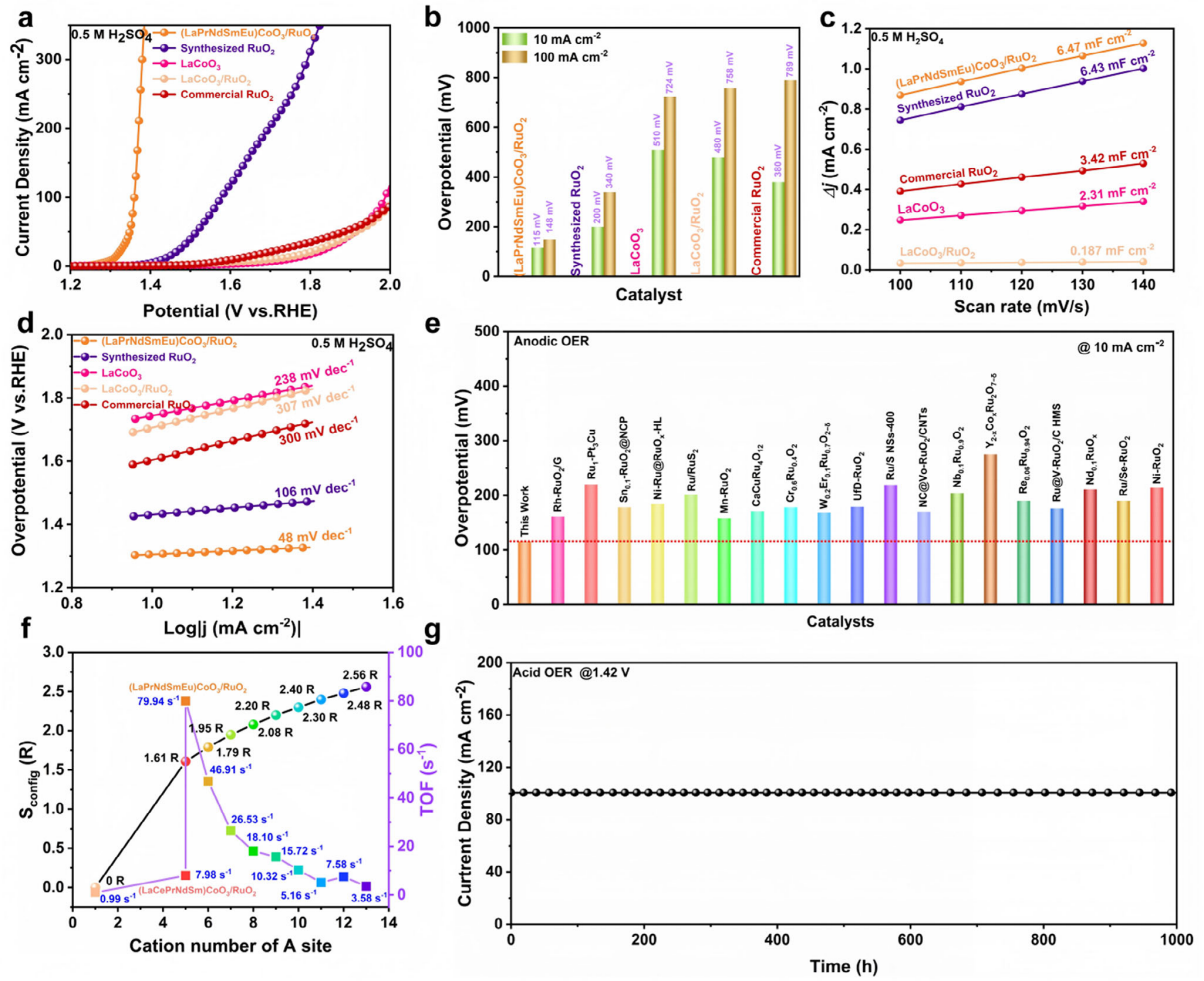
OER performance of catalyst with other counterparts in 0.5 M H_2_SO_4_ electrolyte. (a) OER LSV, (b) overpotential at 10 and 100 mA cm^−2^, (c) capacitive current densities and (d) Tafel of synthesized RuO_2_, commercial RuO_2_, LaCoO_3_, LaCoO_3_/RuO_2_ and (LaPrNdSmEu)CoO_3_/RuO_2_. (e) Comparison of the overpotential at the current density of 10 mA cm^−2^ of (LaPrNdSmEu)CoO_3_/RuO_2_ with recently reported Ru‐based OER electrocatalysts. (f) The dependence of S_config_ and TOF values. (g) Chronopotentiometric curves of acid OER at 100 mA cm^−2^.

The electrochemical activity of the prepared catalysts was evaluated over a wide pH range by a typical three‐electrode test system. The OER activities of a series of HERE perovskite oxides without RuO_2_ in the full pH range were tested first, and the results showed that the high entropy effect significantly improved the activity of the catalyst (Figure S17). The OER performance of LaCoO_3_, LaCoO_3_/RuO_2_, (LaPrNdSmEu)CoO_3_/RuO_2_, commercial RuO_2,_ and synthesized RuO_2_ in the acid condition was displayed in Figure 4a,b. Among them, (LaPrNdSmEu)CoO_3_/RuO_2_ exhibits optimal OER performance with overpotentials of 115 and 148 mV to achieve the current density of 10 and 100 mA cm^−2^, which is much lower than those of LaCoO_3_ (510 and 740 mV), LaCoO_3_/RuO_2_ (480 and 758 mV), commercial RuO_2_ (380 and 789 mV), and synthesized RuO_2_ (200 and 340 mV) under the same conditions. (LaPrNdSmEu)CoO_3_/RuO_2_ exhibits the highest mass activity (18103.3 A g^−1^_Ru_) compared to other control samples (Figure S18) [48, 49, 50]. The effect of different amounts of Ru on the activity was considered in the alkaline environment. The results indicate that the catalyst exhibits optimal activity when the mass ratio of LaCoO_3_ to RuO_2_ is 3:1, which also remains valid under neutral conditions. (For the sake of discussion, LaCoO_3_/RuO_2_ (3:1) was denoted as LaCoO_3_/RuO_2_) (Figure S19). Electrochemical double layer capacitance (C_dl_) results also show that (LaPrNdSmEu)CoO_3_/RuO_2_ has the largest electrochemically active surface area (6.47 mF cm^−2^) compared to LaCoO_3_ (2.31 mF cm^−2^), LaCoO_3_/RuO_2_ (0.187 mF cm^−2^), commercial RuO_2_ (3.42 mF cm^−2^) and synthesized RuO_2_ (6.43 mF cm^−2^), which is also consistent with the results of linear sweeping vloltammetry LSV (Figure 4c; Figure S20). In addition to C_dl_, the Tafel slope was also used to evaluate the reaction kinetics of the prepared catalysts. Compared with LaCoO_3_ (238 mV dec^−1^), LaCoO_3_/RuO_2_ (307 mV dec^−1^), commercial RuO_2_ (300 mV dec^−1^) and synthesized RuO_2_ (106 mV dec^−1^), (LaPrNdSmEu)CoO_3_/RuO_2_ has the fastest reaction kinetics with Tafel slope of only 48 mV dec^−1^, which is consistent with the result that (LaPrNdSmEu)CoO_3_/RuO_2_ has the best OER activity (115 mV@10 mA cm^−2^) (Figure 4d). Even compared to recently reported catalysts of the same type, the OER performance of the prepared catalyst still shows a significant advantage (Figure 4e; Table S3). As shown in Figure 4f and Figure S21, the dependence of S_config_ and the turnover frequency (TOF) values of various catalysts further confirm that there is a strong correlation between the structure and activity of the catalyst. TOF values of (LaCePrNdSm)CoO_3_/RuO_2_ and (LaPrNdSmEu)CoO_3_/RuO_2_ are 7.98 and 79.94 s^−1^, respectively, and the observed discrepancy can be attributed to structural differences between (LaCePrNdSm)CoO_3_/RuO_2_ (Figure 2a) and (LaPrNdSmEu)CoO_3_/RuO_2_ (Tables S4 and S5). The intrinsic activity of the catalyst decreases with the increase of its entropy, which may be caused by excessive lattice distortion and reduced lattice oxygen defects. Electrochemical impedance spectroscopy (EIS) plots of the prepared samples are shown in Figure S22, in which the lowest impedance of (LaPrNdSmEu)CoO_3_/RuO_2_ indicates that it follows favorable reaction kinetics. In addition, the EIS spectra of perovskite oxides with different entropy values in acidic environments show that their conductivity slightly improves with the increase of system entropy, consistent with the Tafel results (Figure S23). Besides the activity of the catalyst, the stability of the reaction is another important index used to evaluate the performance of the catalyst. The *i‐t* curve of (LaPrNdSmEu)CoO_3_/RuO_2_ shows that it can work stably at 100 mA cm^−2^ for more than 1000 h without significant attenuation, which indicates that the strategy of high entropy and heterogeneous interface construction can ensure the long‐term reactivity and stability of the catalyst (Figure 4g). Furthermore, in an actual proton exchange membrane water electrolysis (PEMWE) system, the cell employing (LaPrNdSmEu)CoO_3_/RuO_2_ as the anode exhibited outstanding performance at 50°C in 0.5 M H_2_SO_4_. A cell voltage of only 1.68 V was required to achieve a current density of 1.0 A cm^−2^, and it operated stably at this current density for over 200 h. This performance is also significantly superior to that of recently reported PEMWE systems with analogous catalyst structures (Figure S24). The electrochemical activity of a series of high‐entropy catalysts with heterogeneous structures over a wide pH range has been evaluated. As displayed in Figures S25–S27, the (LaPrNdSmEu)CoO_3_/RuO_2_ requires only overpotentials of 115, 340, and 223 mV to achieve a current density of 10 mA cm^−2^ in 0.5 M H_2_SO_4_, 1.0 M KOH, and 1.0 M PBS. It is worth noting that the prepared series of catalysts shows the best activity in an acidic environment, followed by a neutral environment, which is better than the activity in an alkaline environment, indicating that the activity of such catalysts is strongly dependent on the pH of the electrolyte. Surprisingly, the prepared catalyst also showed excellent HER activity in an alkaline environment, requiring only 77 mV to reach a current density of 10 mA cm^−2^ (Figure S28). In view of the excellent electrochemical activity of the catalyst, it is also very important to investigate the relationship between its structure and its intrinsic activity. The analysis of C_dl_ and EIS under alkaline conditions confirmed this conclusion (Figures S29 and S30).

**FIGURE 5:**
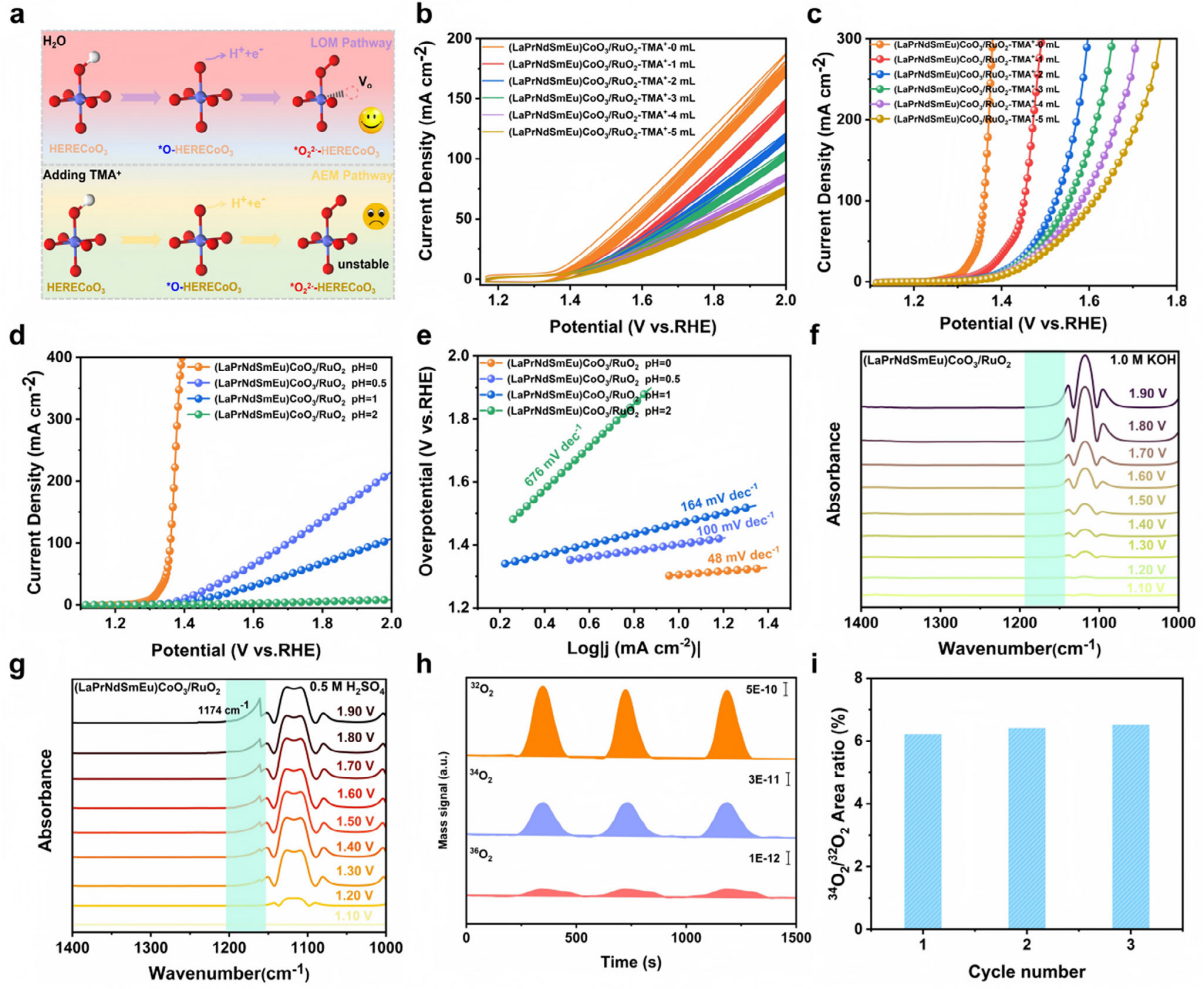
(a) Schematic illustration of intermediate model in CV peaks with and without TMA^+^. (b) CV curves of (LaPrNdSmEu)CoO_3_/RuO_2_ with different concentrations of TMA^+^. (c) LSV curves of (LaPrNdSmEu)CoO_3_/RuO_2_ with different concentrations of TMA^+^. (d) pH dependence of the OER activities of (LaPrNdSmEu)CoO_3_/RuO_2_. (e) The pH dependence of the Tafel value of (LaPrNdSmEu)CoO_3_/RuO_2_. *In‐situ* ATR‐IR spectra of (LaPrNdSmEu)CoO_3_/RuO_2_ in (f) 1.0 M KOH and (g) 0.5 M H_2_SO_4_ solution. (h) DEMS profiles using ^18^O‐labeled catalysts showing generation of ^32^O_2_, ^34^O_2,_ and ^36^O_2_ during potential pulsing cycles. (i) Isotopic oxygen ratio (^34^O_2_/^32^O_2_) over multiple cycles.

In order to further study the reaction mechanism of the catalyst, tetramethylammonium (TMA^+^) was dropped slowly into the electrolyte under the same conditions. Since ^*^O_2_^2^^−^ can be combined with TMA^+^, the kinetics of OER can be reduced through the LOM pathway, according to which the reaction mechanism can be determined as AEM or LOM (Figure 5a) [19, 51, 52, 53]. As shown in Figure 5b,c, with the concentration of TMA^+^ gradually increasing, the current density in both the cyclic voltammetry and the LSV curve of (LaPrNdSmEu)CoO_3_/RuO_2_ gradually decreases, which indicates that the combination of TMA^+^ and ^*^O_2_^2−^ inhibits the OER kinetics through the LOM pathway. Throughout the full cyclic voltammetry (CV) cycling process, it is evident that synthesized RuO_2_ and (LaPrNdSmEu)CoO_3_/RuO_2_ exhibit significant responses to TMA^+^ ions. This indicates that, compared to Co sites, Ru serves as the primary water dissociation site in acidic environments, generating a higher concentration of OH^−^. Furthermore, as the number of RE elements at the La sites increases, the spin state of the Co sites transitions from LS to IS. Correspondingly, the TMA^+^ response signal becomes more pronounced, confirming that an increase in system entropy not only induces regulation of the Co spin state through oxygen vacancies but also enhances the adsorption of OH^−^ at both Co and Ru active sites, thereby compensating for the structural defects associated with the LOM (Figures S31 and S32). In general, OER processes involving lattice oxygen exhibit typical pH‐dependent activity, mainly due to the difference between electron transfer kinetics and proton transfer kinetics leading to this phenomenon [54]. Moreover, (LaPrNdSmEu)CoO_3_/RuO_2_ exhibits significant pH‐dependent activity in acidic environments, which indicates that the catalyst follows a special coordinated proton‐electron transfer (CPET) LOM pathway during OER (Figure 5d,e). In situ attenuated total reflection infrared (ATR‐IR) spectrum was used to gain insight into the LOM (Figure 5f,g) [55, 56, 57]. Under acid measurement conditions, the characteristic absorption peak at 1174 cm^−1^ can be observed in the spectrum of (LaPrNdSmEu)CoO_3_/RuO_2_. When the applied potential was increased to the OER reaction region (≥ 1.4 V vs. RHE), the intensity of the characteristic peak also increased significantly, indicating that abundant reaction intermediates were generated. The absorption peak at 1174 cm^−1^ corresponds to the O─O vibration of the negatively charged peroxide, which is an intermediate of LOM (Figure S33) [58, 59]. To provide direct evidence of lattice oxygen participation, differential electrochemical mass spectrometry (DEMS) analysis was conducted (Figure 5h,i). After the addition of H_2_^18^O and multiple cycles of CV electrochemical pretreatment, dynamic surface reconstruction and the incorporation of ^18^O into the lattice of (LaPrNdSmEu)CoO_3_/RuO_2_ were induced. Subsequent CV measurements performed in H_2_^16^O electrolyte revealed periodic signals of ^16^O^18^O (m/z = 34), confirming the activation of lattice oxygen [60, 61].

Based on the above electrochemical test and catalyst characterization results, a possible catalytic reaction mechanism was proposed. LaCoO_3_/RuO_2_ exhibited unsatisfactory OER activity and electrochemical stability in acidic environments (Figure S34). In these special heterostructures, RuO_2_ was generally considered to have excellent hydrolytic dissociation activity, but its poor reaction stability as an active site limits its wide application. (LaPrNdSmEu)CoO_3_/RuO_2_ with high activity and long‐term stability was successfully prepared by using a high entropy strategy for the La position in LaCoO_3_. Compared to LaCoO_3_/RuO_2_ with AEM, the OER pathway of (LaPrNdSmEu)CoO_3_/RuO_2_ follows a highly active LOM, but the stability of this reaction mechanism is not satisfactory. The high entropy effect induces the conversion of the spin state of the octahedral Co center in (LaPrNdSmEu)CoO_3_/RuO_2_ from LS to IS, which can promote the adsorption of oxygen‐containing intermediates at the Co site, alleviate the damage to the catalyst structure caused by the overreaction of the defects of oxygen production in the lattice oxygen machine, and improve the reaction stability of the catalyst. In addition, due to the unique H_2_O dissociation of RuO_2_ in an acidic environment, the concentration of oxygen‐containing species can be effectively maintained, which further ensures that Co with the IS state can improve the stability of the catalyst. The novel spin‐regulated LOM (SR‐LOM) makes up for the poor stability of LOM, and the lattice oxygen concentration in a series of HERECoO_3_/RuO_2_ was regulated by a high entropy strategy, which improves the reaction activity of the catalyst. In addition, the characterization of (LaPrNdSmEu)CoO_3_/RuO_2_ after OER testing was provided for further validation of the reaction mechanism. The SEM results showed that the morphology of the prepared catalyst did not change significantly before and after electrochemical testing (Figure S35a,b). The heterogeneous interface can still be clearly observed in the TEM images of the catalyst after the OER test, and the combination of SEM results further indicates that the catalyst shows excellent reaction stability, which also confirms the importance of SR‐LOM in maintaining the stability of the (LaPrNdSmEu)CoO_3_/RuO_2_ (Figure S35c–e). Furthermore, the O 1s spectrum of (LaPrNdSmEu)CoO_3_/RuO_2_ at different times indicates a transition between oxygen vacancies, lattice oxygen, M─O, and H_2_O during the stability testing process. The M‐^*^O characteristic peak appeared in the O 1s spectrum when the stability test time was 10 h (Figure S36a). This may be due to the increase in the concentration of oxygen‐containing intermediates during the dissociation process of H_2_O, which were captured by the octahedral center Co and formed a Co─O bond [62, 63, 64]. Then, with the extension of the stabilization time, the oxygen species adsorption and reconfiguration of the catalyst make the characteristic peak of the oxygen defect disappear gradually, and the intensity of the lattice oxygen peaks is enhanced, which indicates that the reconstruction of the catalyst has been completed. It should be noted that there is no significant change in the spectra of La 3d, Ru 3d, and Co 2p before and after the stability test, which is consistent with the characteristics of LOM (Figure S36b–d). The disappearance of characteristic peaks may be due to the dissolution of some elements and weakened signals caused by long‐term testing. The ICP‐MS values of all elements in (LaPrNdSmEu)CoO_3_/RuO_2_ and (LaPrNdSmEuGdTbDyHoErTmYbLu)CoO_3_/RuO_2_ after OER cycle testing were used to illustrate the role of the high entropy effect in maintaining the stability period of the catalyst structure, as shown in Tables S8 and S9. The results indicate that under the same conditions, catalysts with higher configuration entropy have a positive effect on suppressing RE ions, Co ions, and Ru ions compared to those with lower configuration entropy. During the long‐term test, the entropy‐stabilized lattice enables reversible conversion between V_O_, lattice oxygen, and M─O species, leading to the stable OER.

**FIGURE 6:**
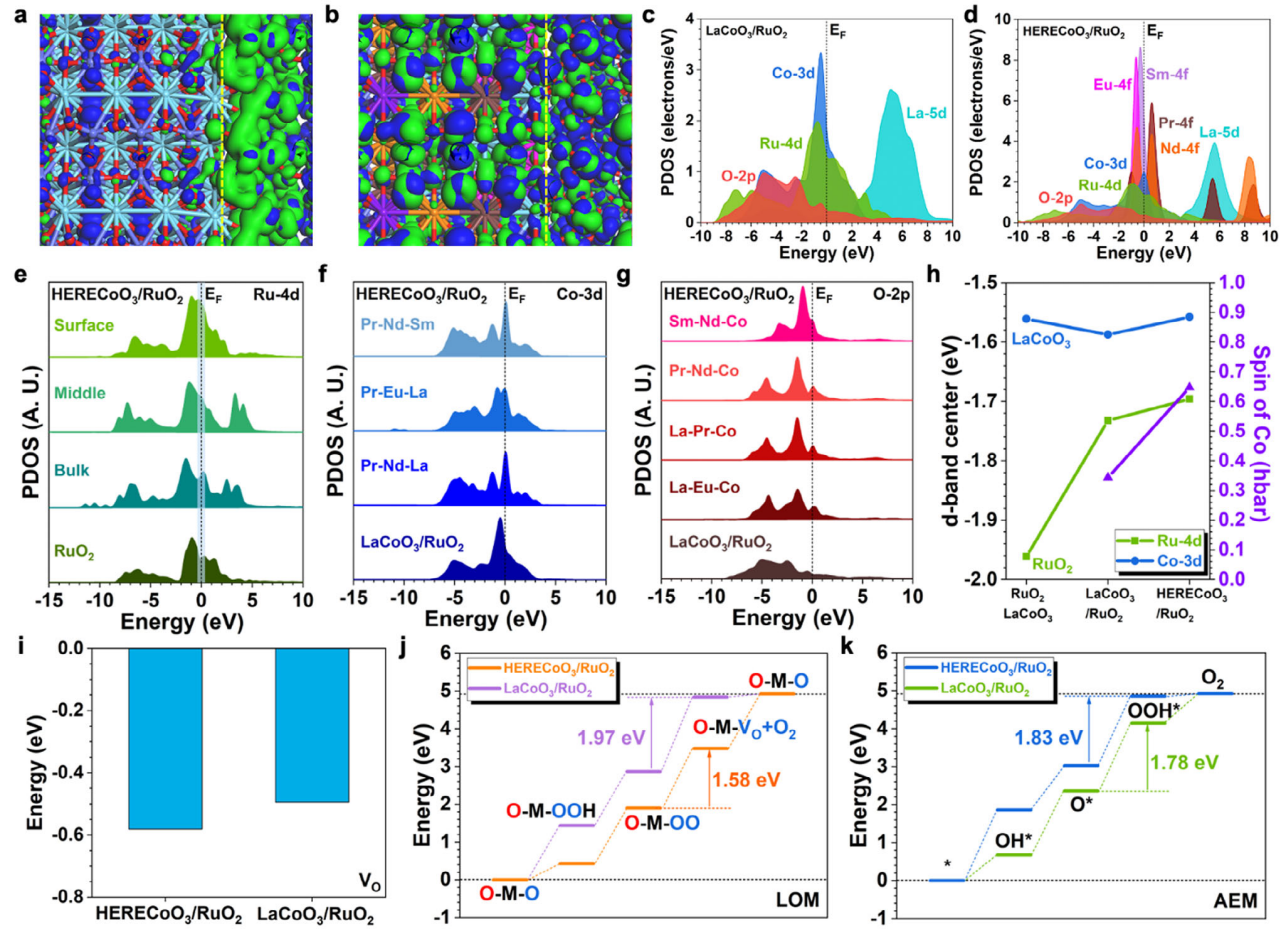
The electronic distributions of bonding and anti‐bonding orbitals near the Fermi level in (a) LaCoO_3_/RuO_2_ and (b) HERECO_3_/RuO_2_. Blue isosurface = bonding orbitals and green isosurface = anti‐bonding orbitals. Green balls = Ru, Light blue balls = La, Blue balls = Co, Brown balls = Pr, Orange balls = Nd, Purple balls = Sm, Pink balls = Eu, and Red balls = O. Yellow dash lines indicate the interface. The PDOS of (c) LaCoO_3_/RuO_2_ and (d) HERECO_3_/RuO_2_. The site‐dependent PDOS of (e) Ru‐4d, (f) Co‐3d and (g) O‐2p in LaCoO_3_/RuO_2_ and HERECO_3_/RuO_2_. (h) The d‐band center variations of Ru‐4d and Co‐3d orbitals. (i) The formation energy of oxygen vacancies in LaCoO_3_/RuO_2_ and HERECO_3_/RuO_2_. The OER reaction trends following (j) LOM and (k) AEM.

To investigate the OER performances of the heterostructures, we have further introduced DFT calculations to reveal the electronic modulations and reaction trend variations induced by the constructions of HERECoO_3_. Notably, the electronic distributions have been evidently changed with the introduction of multi‐RE elements in the La sites in LaCoO_3_ (Figure 6a,b). For the LaCoO_3_/RuO_2_, it is noted that bonding and anti‐bonding orbitals are mainly dominated by the RuO_2_ part as the main active sites, while only LaCoO_3_ contributes limitedly to the bonding orbitals in Co sites. In contrast, the orbital couplings between bonding and anti‐bonding orbitals in HERECO_3_/RuO_2_ become stronger to promote electron transfer within the heterostructures. Moreover, the electroactivity of HERECoO_3_ has also been activated since the bonding orbitals are distributed broadly on the surface of HERECO_3_/RuO_2_. The interface also becomes more electroactive due to the stronger interactions between HERECO_3_ and RuO_2_. The electronic structures also confirm the electronic modulations through the projected partial density of states (PDOS) of the heterostructures. For LaCoO_3_/RuO_2_, it is noted that Co‐3d and Ru‐4d orbitals are both located close to the Fermi level (E_F_) (Figure 6c). The La‐5d orbitals are mainly located above the E_F_ with limited contributions to the electroactivity. The O‐2p orbitals are located at the deep position below the E_F_, displaying good overlap with both Co‐3d and Ru‐4d orbitals to support the stable structures. Although all the RE elements occupy the La sites that are not the active sites for OER, the introduction of unique 4f orbitals has largely modulated the electronic structures in HERECO_3_/RuO_2_ (Figure 6d). The Co‐3d orbitals are upshifted with the main peak located on the E_F_, which is attributed to the stronger interactions induced by the high entropy strategy. More importantly, there are abundant 4f orbitals agglomerate near the E_F,_ including Sm‐4f, Eu‐4f, Nd‐4f, and Pr‐4f, which not only supply fast site‐to‐site electron transfer pathways within HERECO_3_/RuO_2_ but also significantly increase the electron density near the E_F_, leading to more efficient electron transfer during the OER. Although the high entropy introduces evident lattice distortion (AC‐TEM in Figure 2), and typically causes mild charge carrier scattering, the stronger electronic coupling at the interface and agglomeration of RE‐4f orbitals create continuous electron transfer channels, accelerating carrier hopping to offset the influences on the charge carrier mobility. The detailed electronic structures are further compared by site‐dependent PDOS of Ru‐4d, Co‐3d, and O‐2p orbitals. For Ru‐4d orbitals, the formation of heterostructures significantly improves the electron density near the E_F_ from the bulk to the surface in the HERECO_3_/RuO_2_ (Figure 6e). The overall upshifting trend of Ru‐4d orbitals after forming the heterostructures demonstrates the slightly increased valence states of Ru sites, which are consistent with the XPS results. When compared to LaCoO_3_/RuO_2_, we notice the evident upshifting of Co‐3d orbitals to the E_F_ in the HERECO_3_/RuO_2_, indicating the increased electroactivity induced by the electronic modulations by RE elements (Figure 6f). The PDOS of Ru‐4d and Co‐3d orbitals have confirmed that the formation of heterostructure and introduction of high entropy RE elements in La sites are both beneficial for the OER process. The modulations of the O sites are also evident under different surrounding RE elements (Figure 6g). Compared to LaCoO_3_/RuO_2_, the HERE elements have induced obvious upshifts of the O‐2p orbitals on the surface. In particular, the Sm‐Nd‐Co coordinated O sites not only show the highest p orbitals but also enhance the electron density near the E_F_. These electronic modulations slightly narrow the band gap and increase the electron density near the E_F_ to enhance the improved conductivity as characterized by experiments (Figure S23). The high electroactivity of lattice oxygen supports the transition from AEM to LOM in HERECO_3_/RuO_2_ for the efficient OER process. According to the electronic structures, we have also demonstrated the d‐band center evolutions of both Ru and Co sites (Figure 6h). It is noted that the d‐band center of Co and Ru sites displays the converse trend when forming the heterostructures, revealing the electron transfer from RuO_2_ to LaCoO_3,_ as experimental characterizations. With the high entropy RE elements, the valence states of both Ru and Co exhibit an increasing trend. Moreover, the average spin of Co sites also slightly increases as the Co sites vary from low spin to intermediate spin with the high entropy strategy. The spin‐polarized PDOS demonstrates that the original Co‐3d sites display a relatively weak mismatch between the spin‐up and spin‐down states, supporting the low spin state (Figure S37). With the introduction of high entropy elements in La sites, the mismatch between the spin‐up and spin‐down states of Co‐3d orbitals is significantly enlarged, which supports the change from low spin to intermediate spin.

Meanwhile, the high entropy strategy also modulates the electroactivity of the lattice oxygen. Notably, the formation of oxygen vacancies becomes easier with lower average formation energy in HERECoO_3_/RuO_2_, which is calculated on surface sites on the HERECoO_3_, RuO_2_, and interface regions in the heterostructure (Figure 6i). The high‐entropy effect lowers the oxygen vacancy formation energy, which is consistent with the increasing V_O_ concentration with increasing entropy in Figure 3e. This also confirms that lattice oxygens become more flexible to initiate the LOM for OER. Then, we compared the reaction trends for LOM in both LaCoO_3_/RuO_2_ and HERECoO_3_/RuO_2_ (Figure 6j,k). Under the LOM, the rate‐determining step (RDS) occurs at the generation of O_2_ with the formation of oxygen vacancy in the lattice, where HERECoO_3_/RuO_2_ shows a much lower energy barrier of 1.58 eV than the LaCoO_3_/RuO_2_ (1.97 eV). Compared with the LOM, the RDS barrier is enlarged for HERECO_3_/RuO_2_ but slightly reduced for the LaCoO_3_/RuO_2_, indicating the change of OER mechanism preference in HERECoO_3_/RuO_2_. The overpotentials of HERECoO_3_/RuO_2_ are 0.35 and 0.50 V for LOM and AEM, respectively, supporting that HERECoO_3_/RuO_2_ prefers to undergo the LOM to guarantee superior OER performances. In the meantime, the LaCoO_3_/RuO_2_ favors the AEM due to slightly lower overpotential, revealing that the high entropy strategy successfully modulates the OER mechanisms.

## 3 Conclusion

In summary, (HEREs)CoO_3_/RuO_2_ (RE = La, Pr, Nd, Sm, Eu, Gd, Tb, Dy, Ho, Er, Tm, Yb, and Lu) and (LaPrNdSmEu)CoO_3_/RuO_2_ with high OER performance and excellent stability were prepared via a facile calcination‐assisted sol‐gel method. The synthesized (LaPrNdSmEu)CoO_3_/RuO_2_ shows superior OER activity over the wide pH range, needing overpotentials only of 115, 340 and 223 mV to achieve a current density of 10 mA cm^−2^ in 0.5 M H_2_SO_4_, 1.0 M KOH and 1.0 M PBS solutions, even to obtain a current density of 400 mA cm^−2^, only an overpotential of 163 mV is required. Besides, the prepared catalyst still possesses excellent HER activity in an alkaline environment, requiring only 77 mV to achieve a current density of 10 mA cm^−2^. Moreover, (LaPrNdSmEu)CoO_3_/RuO_2_ not only has excellent electrochemical stability (stable operation for 1000 h at a current density of 100 mA cm^−2^), but also shows extremely high electrochemical intrinsic activity (TOF value of 79.94 s^−1^ at an overpotential of 150 mV). The results of AC‐TEM, EELS, and in situ ATR‐IR showed that the high entropy strategy of RE elements in LaCoO_3_ regulated the OER mechanism, transforming the traditional AEM into highly active LOM, and the high entropy effect can also induce the spin state of the octahedral Co from the LS to IS, which promotes the adsorption of Co to oxygen‐containing intermediates and makes up for the poor stability of LOM. The novel SR‐LOM achieves a win‐win situation of high activity and long‐term stability of the catalyst. DFT calculations have indicated that the high entropy strategy will induce evident electronic modulations in the HERECO_3_/RuO_2_, in which the electroactivity of Ru sites is enhanced and the spins of Co sites are increased. The stronger interactions at the heterointerfaces of HERECO_3_/RuO_2_ activates the lattice oxygen and modulates the AEM in LaCO_3_/RuO_2_ to the LOM in HERECO_3_/RuO_2_, leading to improved OER performances in different environments. This work provides a new perspective for the design and study of RE‐based water electrolysis catalysts in the future.

## Conflicts of Interest

The authors declare no conflicts of interest.

## Supporting information




**Supporting File**: adma73021‐sup‐0001‐SuppMat.docx.

## Data Availability

The data that support the findings of this study are available from the corresponding author upon reasonable request.
