# Supplementary material for: Entropy‐Driven Spin Transition in Rare Earth Perovskites Enables Feedback Adsorption for Enhanced Acidic Water Oxidation
Source: Adv Mater. 2026 Apr 8;38(26):e73021. doi: 10.1002/adma.73021 (PMC13155283; doi:10.1002/adma.73021)
Supplement: Supplementary file 1 — Supporting File: adma73021‐sup‐0001‐SuppMat.docx. [file ADMA-38-e73021-s001.docx]

Supporting Information

Entropy-Driven Spin Transition in Rare Earth Perovskites Enables Feedback Adsorption for Enhanced Acidic Water Oxidation

Yong Jiang, Mingzi Sun, Zhong Liang, Hao Fu, Ziyun Zhong, Bolong Huang,^*^ Yaping Du^*^

**Experimental Details**

***Chemicals and materials***

Dipotassium hydrogen phosphate (KH_2_PO_4_), Potassium dihydrogen phosphate (K_2_HPO_4_), Citric acid monohydrate (C_6_H_10_O_8_), Urea (CO(NH_2_)_2_), Cobalt(II) acetate tetrahydrate ((CH_3_COO)_2_Co·4H_2_O) and potassium hydroxide (KOH) were purchased from Shanghai Aladdin Biochemical Technology Co. Ltd.. Lanthanum nitrate (La(NO_3_)_3_), Cerium nitrate (Ce(NO_3_)_3_), Praseodymium nitrate (Pr(NO_3_)_3_), Neodymium nitrate (Nd(NO_3_)_3_), Samarium nitrate (Sm(NO_3_)_3_), Europium nitrate (Eu(NO_3_)_3_), Gadolinium nitrate (Gd(NO_3_)_3_), Terbium nitrate (Tb(NO_3_)_3_), Dysprosium nitrate (Dy(NO_3_)_3_), Holmium nitrate (Ho(NO_3_)_3_), Thulium nitrate (Tm(NO_3_)_3_), Lutetium nitrate (Lu(NO_3_)_3_) were purchased from Beijing Huawei Ruike Chemical Co. Ltd.. Yttrium nitrate (Y(NO_3_)_3_), Erbium nitrate (Er(NO_3_)_3_), Ytterbium nitrate (Yb(NO_3_)_3_) and Sodium borohydride (NaBH_4_) were purchased from Shanghai Macklin Biochemical Co. Ltd.. Ruthenium dioxide (RuO_2_) and Nafion (5 wt%) were purchased from Sigma Aldrich chemical reagent. Ruthenium chloride (RuCl_3_) was purchased from Shanghai Bide Pharmaceutical Technology Co. Ltd.. Doubly distilled deionized water (18.2 MΩ) was used for all experiments. All reagents were directly used without further purification.

***Synthesis of LaCoO_3_/RuO_2_***

In a typical synthetic process, 30 mL of DI water containing 3.75 mmol of Co(OAc)_2_·4H_2_O, 3.75 mmol of La(NO_3_)_3_·6H_2_O, 15 mmol of citric acid monohydrate, 15 mmol of urea, and 3 mL of nitric acid were magnetically stirred to form a homogeneous solution. Then, the mixed solution was heated to 110 °C and kept magnetically stirred to generate a gel. To remove residual water, the as-obtained gel was dried at 130 °C for 12 h. Pristine LaCoO_3_ was finally obtained via calcining the as-obtained gel under air atmosphere at 600 °C for 6 h*.* The as-prepared LaCoO_3_ (30 mg) was dispersed in 100 mL of deionized water containing 10 mg of RuCl_3_ and sonicated for 30 min to obtain a uniform solution. Subsequently, a NaBH_4_ solution (1 mg/mL) was slowly added dropwise. After the solution gradually clarified, it was centrifuged and dried. Then calcined it in the Muffle furnace at 600 °C for 3h, the sample is recorded as LaCoO_3_/RuO_2_ (3:1) after cooling. LaCoO_3_/RuO_2_ (5:1), LaCoO_3_/RuO_2_ (2:1) and LaCoO_3_/RuO_2_ (1:1) obtained by changing the amount of Ru under the same conditions.

***Synthesis of*** ***HERECoO_3_/RuO_2_***

The synthesis process of HERECoO_3_/RuO_2_ is completely consistent with that of LaCoO_3_/RuO_2_. It is worth noting that equal moles of rare earth nitrates were used to replace the La element in A site of LaCoO_3_/RuO_2_ and maintaining a consistent total molar amount. The synthesis process of HERECoO_3_/RuO_2_ is exactly the same as that of LaCoO_3_/RuO_2_, and it is worth noting that the A-site La element is replaced by equal molar of rare earth nitrate, maintaining the same total molar amount.

***Preparation of working electrode***

5 mg catalyst was dispersed in a mixture of 200 µL ultrapure water, 280 µL ethanol, and 20 µL Nafion solution by sonication for 30 min to obtain catalyst ink. For each test, 50 µL catalyst ink was dropped on the precleaned carbon paper electrode (area: 1×1 cm^2^, the loading of the catalysts: 50 ug cm^-2^) and dried in air by natural evaporation.

***Material characterization***

Powder X-ray diffraction (XRD) was performed on Smart-Lab (Rigaku) X-ray diffractometer equipped with graphite monochromaticized Cu Kα radiation (λ = 1.54056 Å). Scanning electron microscopy (SEM) and transmission electron microscopy (TEM) were employed on JEOL JSM-7800F and HT-7800 (Hitachi), respectively. High-resolution TEM (HRTEM) and scanning TEM energy-dispersive X-ray spectroscopy (STEM-EDS) was recorded on JEOL JEM-2800 with an acceleration voltage of 200 kV. [Aberration Corrected Transmission Electron Microscope](http://less.nankai.edu.cn/lims/!equipments/equipment/index.578) was recorded on JEM-ARM200F with an acceleration voltage of 200 kV. X-ray photoelectron spectroscopy (XPS) was conducted on ESCALab 250Xi (Thermo Scientific) X-ray photoelectron spectrometer using Al Kα radiation exciting source. Inductively coupled plasma-optical emission spectrometry (ICP-OES) was determined by ICPS-8100 spectrometer (Shimadzu). X-ray absorption spectroscopy (XAS) on Cu K-edge was performed with Si (111) crystal monochromators at BL11B beamline of Shanghai Synchrotron Radiation Facility (SSRF), and recorded in fluorescence mode and further processed and analyzed by Athena and Artemis software codes.

***XPS analysis of O 1s***

***i) Data preprocessing***

Charge correction: Use the common carbon pollution peak (C 1s, usually set at 284.8 eV) or known internal standards (such as metallic peaks) on the sample surface for binding energy correction.

Background deduction: Shirley background or linear background is usually used. Shirley background is more commonly used because it considers the energy loss of inelastic scattered electrons.

***ii) Identify and define peak components***

A typical metal oxide O 1s spectrum typically contains one or more of the following components, arranged in descending order of binding energy:

Lattice oxygen position (BE): typically in the range of 529.0-530.5 eV; Surface adsorbed oxygen/hydroxyl oxygen/defect oxygen positions (BE): typically in the range of 531.0-532.5 eV; Adsorbed water molecules/physically adsorbed oxygen sites (BE): typically above 533.0 eV.

***iii) Fitting parameter settings***

Peak shape function: Typically, a symmetric Gaussian Lorentzian mixture function is used. This can better reflect the actual shape of XPS peaks.

Half width: Components with the same chemical state should have a similar half width. Usually, the main peak (lattice oxygen) is first fitted and its FWHM is used as a constraint to apply to other peaks belonging to "oxygen" but with different environments (such as surface hydroxyl groups), but allowing for small fluctuations.

***Electrochemical Measurements***

All electrochemical measurements were conducted at room temperature using an electrochemical workstation (CHI660E, CH Instruments). The prepared carbon paper was used as working electrode, a carbon rod as the counter electrode, and a Ag/AgCl electrode (acidic electrolyte) or Hg/HgO (alkaline electrolyte) electrode as the reference electrode. All the potentials reported in this work were converted to the reversible hydrogen electrode (RHE). The potentials were converted to the RHE scale according to the following equation: $E_{RHE}= E_{Hg/HgO}+0.0591\times pH+ E_{Hg/HgO}^{*}$ (in alkaline media), $E_{RHE}= E_{Ag/AgCl}+0.0591\times pH+ E_{Ag/AgCl}^{*}$ (in neutral and acid mediums). All polarization curves were carried out in 0.5 M H_2_SO_4_, 1.0 M KOH and 1.0 M PBS solutions after a continuous cyclic voltammetry, respectively If there were no special cases, the polarization curves were carried out at the scanning rates of 5 mV s^-1^. iR correction was carried out during the test with the values of 95% owing to certain resistance between catalyst and electrolyte. The electrochemical active surface area (ECSA) was measured by electrochemical double electrode method. The double layer capacitance curve (C_dl_) was obtained by cyclic voltammetry scanning at different scanning rates (100-140 mV s^-1^) in non-faradic potential range. The turnover frequency (TOF) of the catalyst active site can be calculated from the following formula: $TOF=jA/mFn$.

***Calculation Setup***

In this work, we have introduced the density functional theory (DFT) calculations through the CASTEP packages to investigate the OER performances of LaCO_3_/RuO_2_ and HERECO_3_/RuO_2_.^[1]^ In particular, the generalized gradient approximation (GGA) and Perdew-Burke-Ernzerhof (PBE) functionals are selected since they are suitable to describe the exchange-correlation interactions.^[2-4]^ Due to current computation resources limitations, the GGA+U methods have not been applied, which will be considered in future works to supply more accurate demonstrations of electronic structures. In the meantime, the cutoff energy has been set to 480 eV, which is automatically generated based on the selection of ultrafine quality and the ultrasoft pseudopotentials. Considering the balance between the computational loading and the calculation efficiency, we have applied the coarse quality of the k-point in 2×2×1 density with 0.05 1/Å separation in the Monkhost-Pack grid with Broyden-Fletcher-Goldfarb-Shannon (BFGS) algorithm for all energy minimization processes.^[5]^ The spin-polarized calculations are also considered in this work for all the electronic structures of different metals.

In this work, the heterostructures of LaCO_3_/RuO_2_ are constructed between the (101) surfaces of RuO_2_ and (012) surfaces of LaCO_3_ with around four-layer thickness. The high entropy strategy of HERECO_3_/RuO_2_ is applied by replacing La sites with Pr, Nd, Sm, and Eu atoms evenly with a composition including 5 La, 5 Pr, 5 Nd, 5 Sm, 4 Eu, and 24 Co atoms in HERECO_3_. We have introduced 20 Å vacuum space in the z-axis for all the models to guarantee sufficient space for geometry optimizations. To accomplish the geometry optimizations, the following convergence criteria are applied including Hellmann-Feynman forces should not exceed 0.001 eV/Å, and the total energy difference should not be over 5×10^-5^ eV/atom for all the calculations.

**Figures and Tables:**





**Figure S1.** XRD pattern of LaCoO_3_ under different temperature treatment.


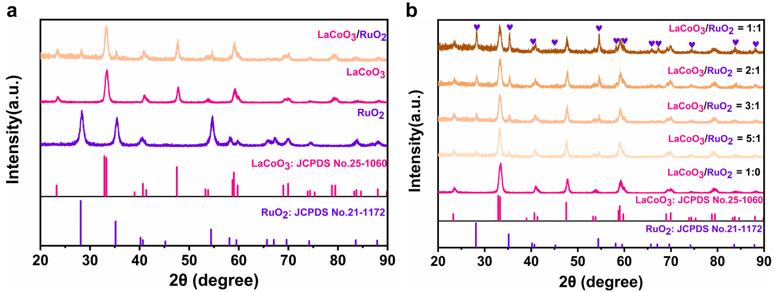


**Figure S2.** (a) XRD patterns of LaCoO_3_/RuO_2_, LaCoO_3_ and RuO_2_. (b) XRD patterns of LaCoO_3_/RuO_2_ with different ratio of LaCoO_3_ and RuO_2_.


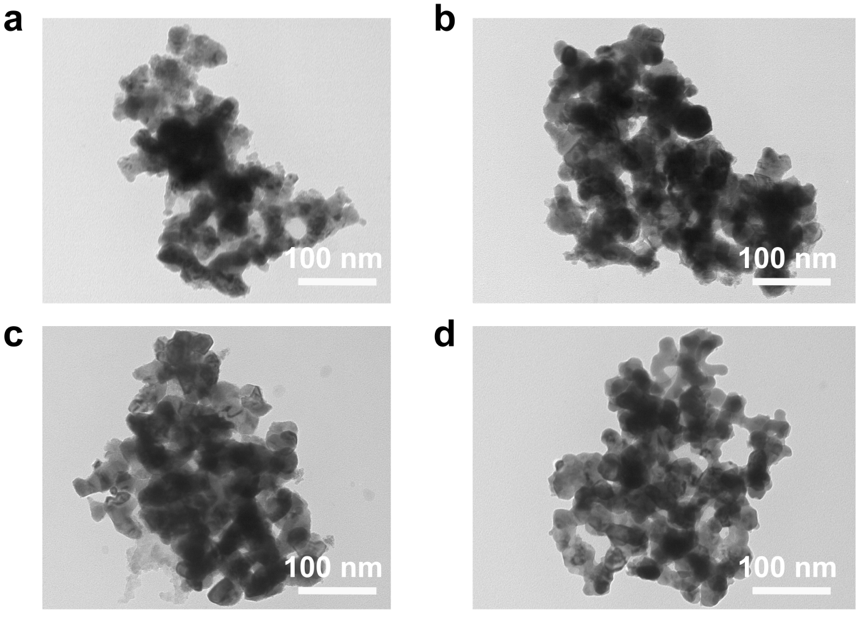


**Figure S3.** TEM images of (a) LaCoO_3_/RuO_2_ (5:1), (b) LaCoO_3_/RuO_2_ (3:1), (c) LaCoO_3_/RuO_2_ (2:1) and (d) LaCoO_3_/RuO_2_ (1:1).


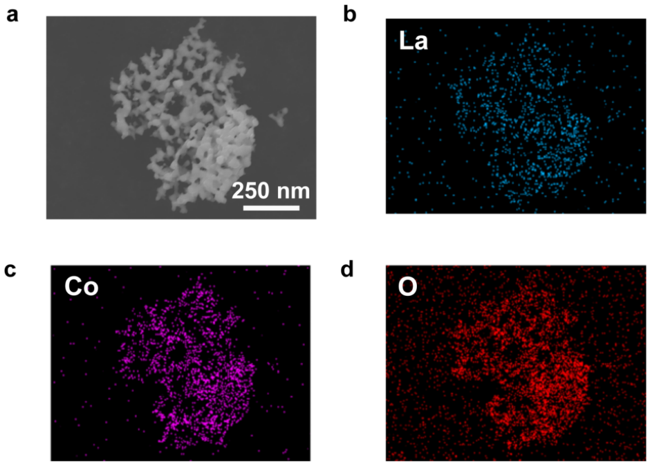


**Figure S4.** SEM image and mapping results of LaCoO_3_.


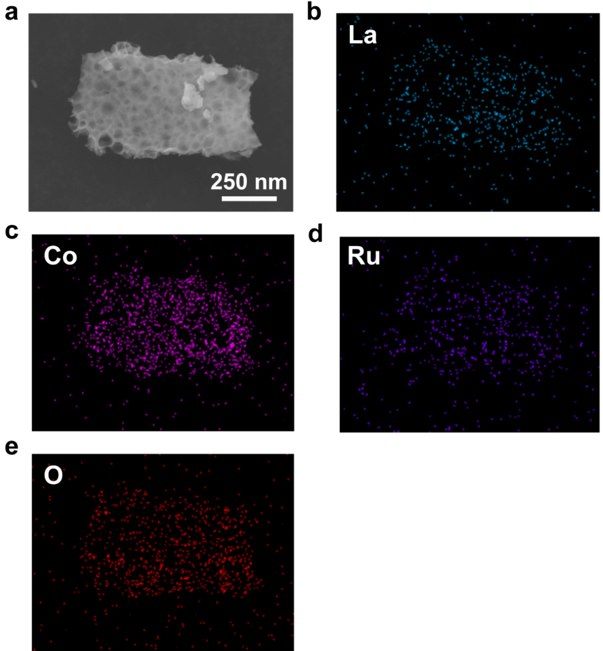


**Figure S5.** SEM image and mapping results of LaCoO_3_/RuO_2_.


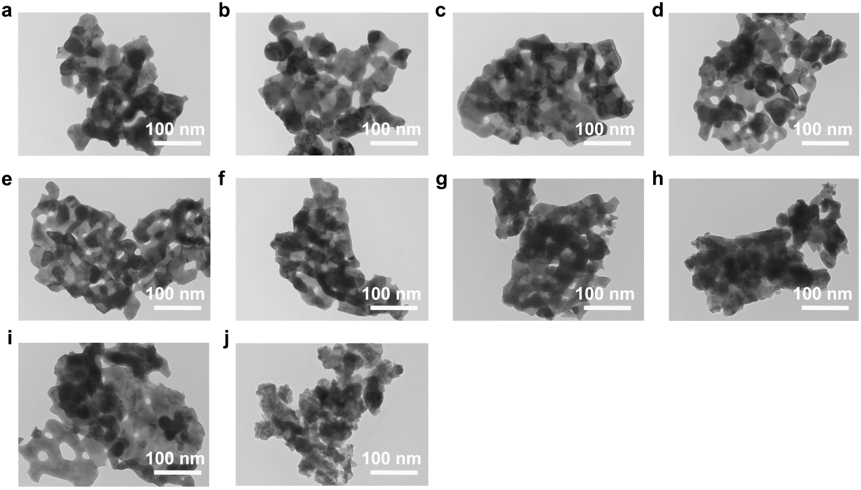


**Figure S6.** TEM images of (a) (LaCePrNdSm)CoO_3_, (b) (LaPrNdSmEu)CoO_3_, (c) (LaPrNdSmEuGd)CoO_3_, (d) (LaPrNdSmEuGdTb)CoO_3_, (e) (LaPrNdSmEuGdTbDy)CoO_3_, (f) (LaPrNdSmEuGdTbDyHo)CoO_3_, (g) (LaPrNdSmEuGdTbDyHoEr)CoO_3_, (h) (LaPrNdSmEuGdTbDyHoErTm)CoO_3_, (i) (LaPrNdSmEuGdTbDyHoErTmYb)CoO_3_, and (j) (LaPrNdSmEuGdTbDyHoErTmYbLu)CoO_3_.





**Figure S7.** XRD pattern of Ru-loaded high entropy perovskite catalysts without air calcination treatment.


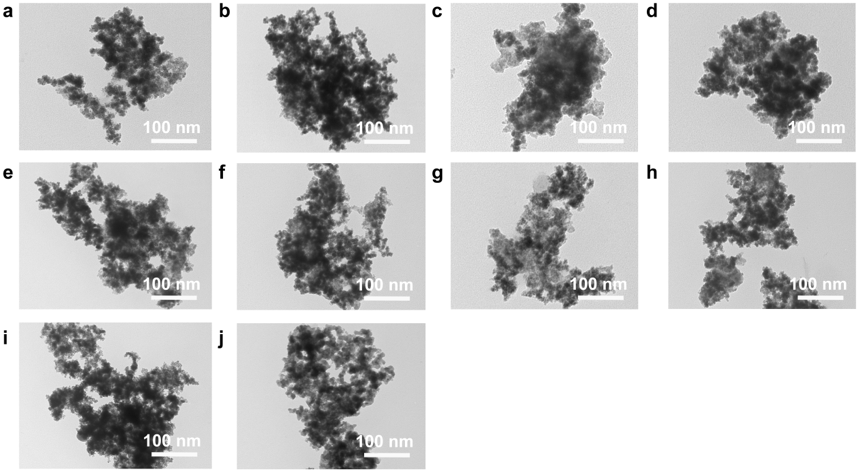


**Figure S8.** TEM images of (a) (LaCePrNdSm)CoO_3_/Ru, (b) (LaPrNdSmEu)CoO_3_/Ru, (c) (LaPrNdSmEuGd)CoO_3_/ Ru, (d) (LaPrNdSmEuGdTb)CoO_3_/Ru, (e) (LaPrNdSmEuGdTbDy)CoO_3_/Ru, (f) (LaPrNdSmEuGdTbDyHo)CoO_3_/Ru, (g) (LaPrNdSmEuGdTbDyHoEr)CoO_3_/Ru, (h) (LaPrNdSmEuGdTbDyHoErTm)CoO_3_/Ru, (i) (LaPrNdSmEuGdTbDyHoErTmYb)CoO_3_/Ru, and (j) (LaPrNdSmEuGdTbDyHoErTmYbLu)CoO_3_/Ru.


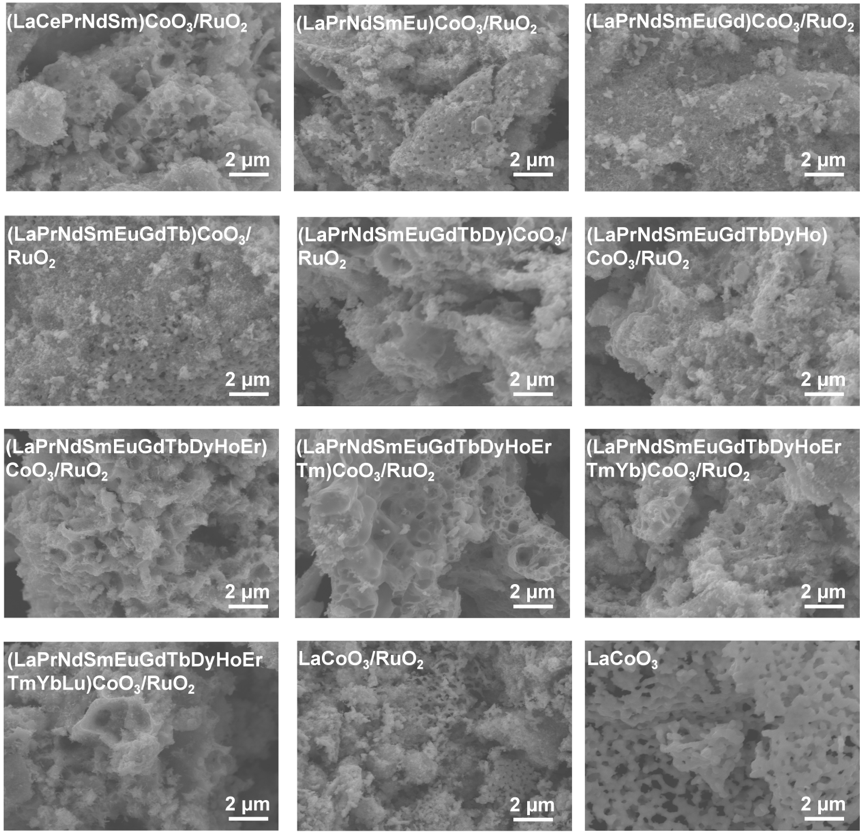


**Figure S9.** SEM images of (HERE)CoO_3_/RuO_2_.


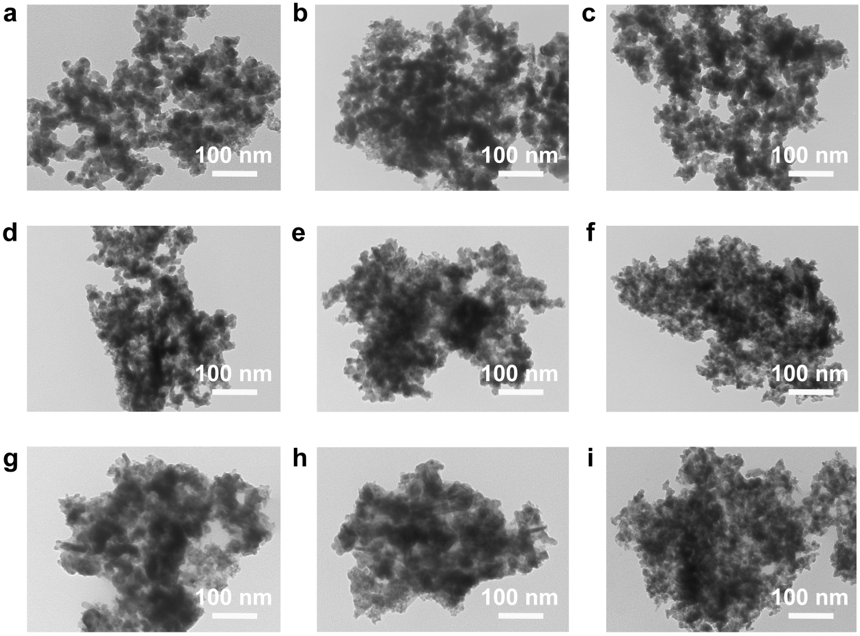


**Figure S10.** TEM images of (a) (LaCePrNdSm)CoO_3_/RuO_2_, (c) (LaPrNdSmEuGd)CoO_3_/RuO_2_ (d) (LaPrNdSmEuGdTb)CoO_3_/RuO_2_, (e) (LaPrNdSmEuGdTbDy)CoO_3_/RuO_2_, (f) (LaPrNdSmEuGdTbDyHo)CoO_3_/RuO_2_, (g) (LaPrNdSmEuGdTbDyHoEr)CoO_3_/RuO_2_, (h) (LaPrNdSmEuGdTbDyHoErTm)CoO_3_/RuO_2_, (i) (LaPrNdSmEuGdTbDyHoErTmYb)CoO_3_/RuO_2_, and (j) (LaPrNdSmEuGdTbDyHoErTmYbLu)CoO_3_/RuO_2_.


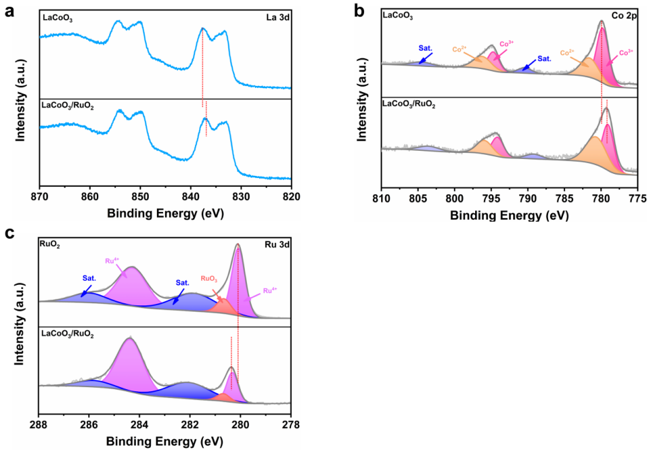


**Figure S11.** XPS results of (a) La 3d, (b) Co 2p and (c) Ru 3d of LaCoO_3_/RuO_2_ and RuO_2_.





**Figure S12.** La 3d spectra of LaCoO_3_, LaCoO_3_/RuO_2_, (LaPrNdSmEu)CoO_3_/RuO_2_, (LaPrNdSmEuGdTbDyHoErTm)CoO_3_/RuO_2_ and (LaPrNdSmEuGdTbDyHoErTmYb)CoO_3_/RuO_2._





**Figure S13.** The EPR spectra of LaCoO_3_, LaCoO_3_/RuO_2,_ (LaPrNdSmEu)CoO_3_/RuO_2_, (LaPrNdSmEuGdTbDyHoErTm)CoO_3_/RuO_2_ and (LaPrNdSmEuGdTbDyHoErTmYb)CoO_3_/RuO_2_.





**Figure S14.** Ru M-edge EELS spectra of LaCoO_3_/RuO_2_ and (LaPrNdSmEu)CoO_3_/RuO_2_.


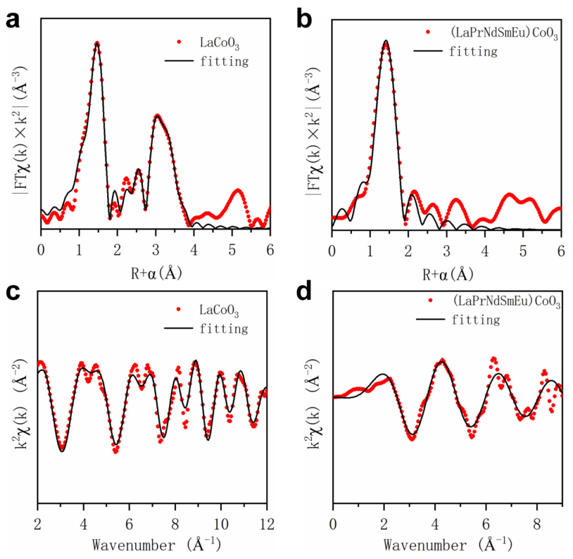


**Figure S15.** (a, b) Fitting results of R space of Co in LaCoO_3_ and (LaPrNdSmEu)CoO_3_. (c, d) Fitting of K space of Co in Co-foil, CoO and Co_3_O_4_.


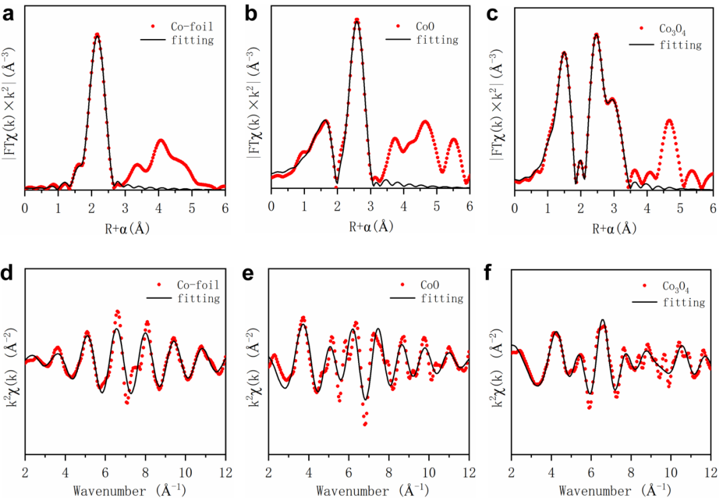


**Figure S16.** (a-c) Fitting results of R space of Co in Co-foil, CoO and Co_3_O_4_. (d-f) Fitting of K space of Co in Co-foil, CoO and Co_3_O_4_.


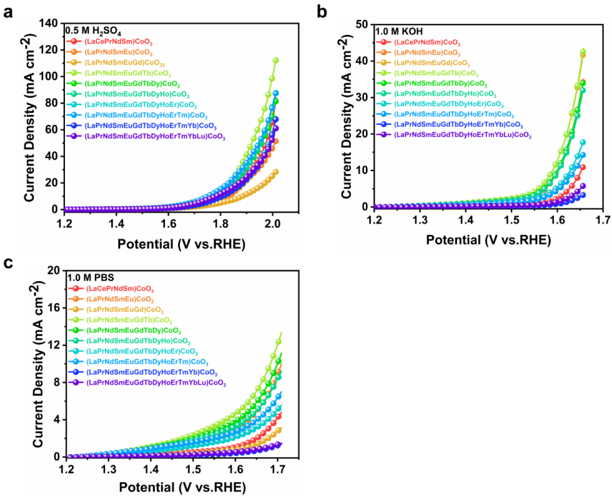


**Figure S17.** LSV polarization curves of (HERE)CoO_3_ for OER in (a) 0.5 M H_2_SO_4_, (b) 1.0 M KOH and (c) 1.0 M PBS solutions.





**Figure S18.** MA of synthesized RuO_2_, commercial RuO_2_, LaCoO_3_/RuO_2_ and (LaPrNdSmEu)CoO_3_/RuO_2_,


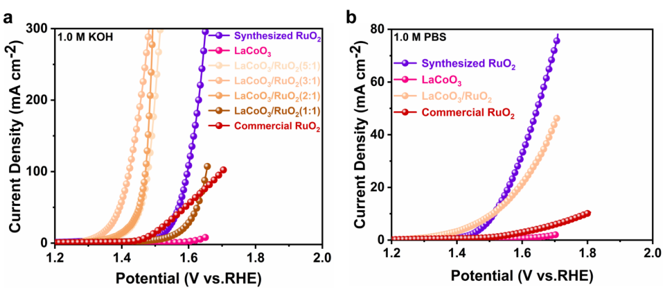


**Figure S19.** (a) LSV polarization curves of synthesized RuO_2_, LaCoO_3_, commercial RuO_2_ and LaCoO_3_/RuO_2_ with different ratio of LaCoO_3_ and RuO_2_ for OER in 1.0 M KOH solution. (b) LSV polarization curves of synthesized RuO_2_, LaCoO_3_, commercial RuO_2_ and LaCoO_3_/RuO_2_ (LaCoO_3_/RuO_2_ (3:1)) for OER in 1.0 M PBS solution.


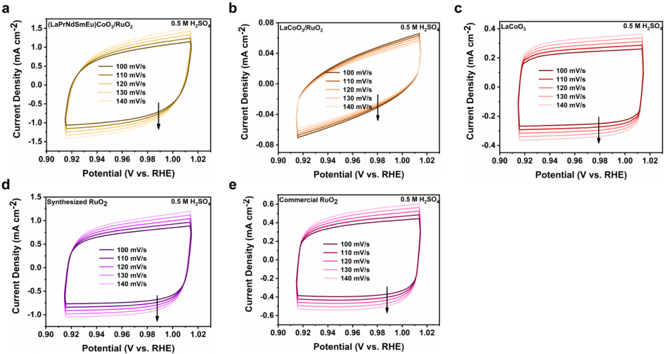


**Figure S20.** Cyclic voltammetry curves for (a) (LaPrNdSmEu)CoO_3_/RuO_2_, (b) LaCoO_3_/RuO_2_, (c) LaCoO_3_, (d) synthesized RuO_2_ and (e) commercial RuO_2_ at different scan rates (100-140 mV s^-1^) for OER in 0.5 M H_2_SO_4_.


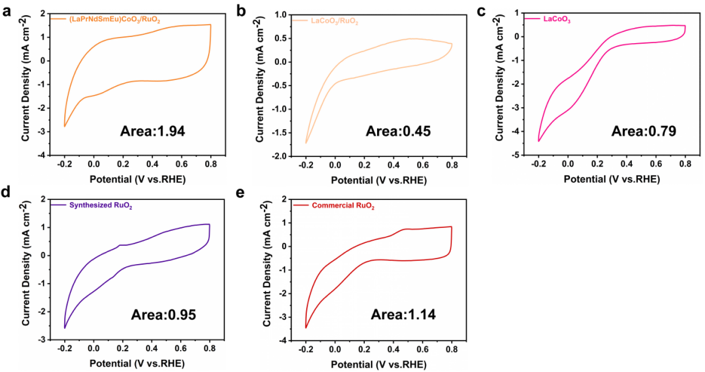


**Figure S21.** Cyclic voltammetry (CV) curves of (a) (LaPrNdSmEu)CoO_3_/RuO_2_, (b) LaCoO_3_/RuO_2_, (c) LaCoO_3_, synthesized RuO_2_ and commercial RuO_2_ were measured at 0.05 V s^-1^ in phosphate buffered saline solution (PBS, pH = 7.0).





**Figure S22.** EIS curves for (LaPrNdSmEu)CoO_3_/RuO_2_, LaCoO_3_/RuO_2_, synthesized RuO_2_, commercial RuO_2_ and LaCoO_3_ in 0.5 M H_2_SO_4_.





**Figure 23.** EIS curves for LaCoO_3_, (LaPrNdSmEu)CoO_3_ and (LaPrNdSmEuGdTbDyHoErTmYbLu)CoO_3_ in 0.5 M H_2_SO_4_.


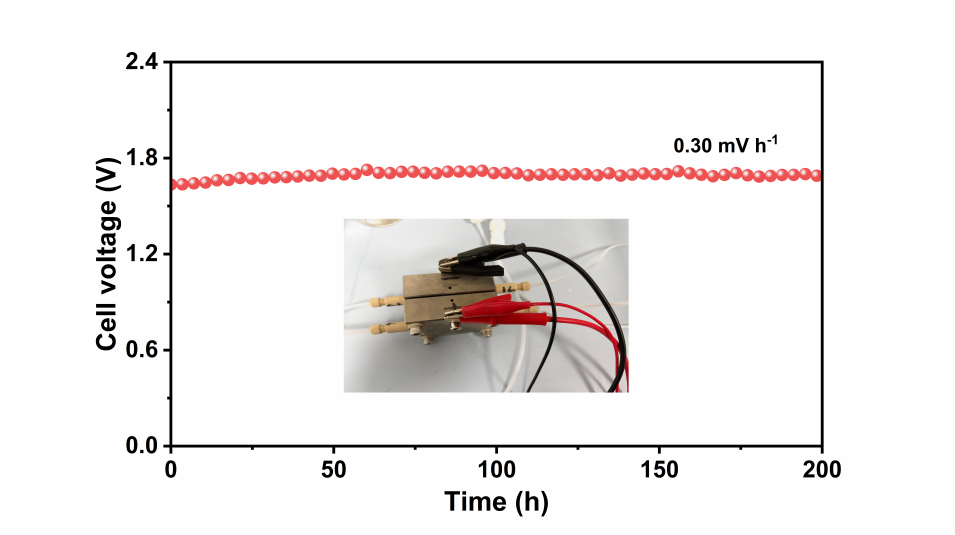


**Figure S24.** Long term durability test of PEMWE electrolysis cell using (LaPrNdSmEu)CoO_3_/RuO_2_ anode at 1.0 A cm^-2^.


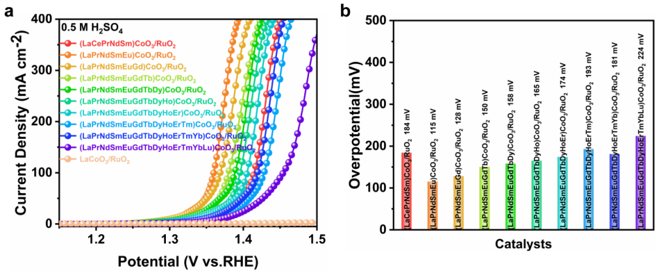


**Figure S25.** (a) OER LSV polarization curves and (b) overpotentials in 0.5 M H_2_SO_4_ at a current density of 10 mA cm^-2^ of synthesized RuO_2_, LaCoO_3_/RuO_2_, (LaCePrNdSm)CoO_3_/RuO_2_ (LaPrNdSmEu)CoO_3_/RuO_2_, (LaPrNdSmEuGd)CoO_3_/ RuO_2_, (LaPrNdSmEuGdTb)CoO_3_/RuO_2_, (LaPrNdSmEuGdTbDy)CoO_3_/RuO_2_, (LaPrNdSmEuGdTbDyHo)CoO_3_/RuO_2_, (LaPrNdSmEuGdTbDyHoEr)CoO_3_/RuO_2_, (LaPrNdSmEuGdTbDyHoErTm)CoO_3_/RuO_2_ (LaPrNdSmEuGdTbDyHoErTmYb)CoO_3_/RuO_2_ and (LaPrNdSmEuGdTbDyHoErTmYbLu)CoO_3_/RuO_2_.


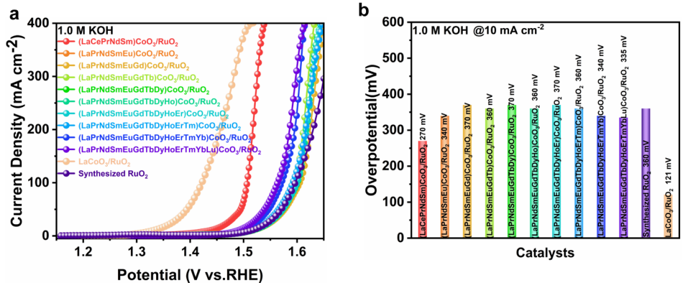


**Figure 26.** (a) OER LSV polarization curves and (b) overpotentials in 1.0 M KOH at a current density of 10 mA cm^-2^ of synthesized RuO_2_, LaCoO_3_/RuO_2_, (LaCePrNdSm)CoO_3_/RuO_2_ (LaPrNdSmEu)CoO_3_/RuO_2_, (LaPrNdSmEuGd)CoO_3_/ RuO_2_, (LaPrNdSmEuGdTb)CoO_3_/RuO_2_, (LaPrNdSmEuGdTbDy)CoO_3_/RuO_2_, (LaPrNdSmEuGdTbDyHo)CoO_3_/RuO_2_, (LaPrNdSmEuGdTbDyHoEr)CoO_3_/RuO_2_, (LaPrNdSmEuGdTbDyHoErTm)CoO_3_/RuO_2_ (LaPrNdSmEuGdTbDyHoErTmYb)CoO_3_/RuO_2_ and (LaPrNdSmEuGdTbDyHoErTmYbLu)CoO_3_/RuO_2_.


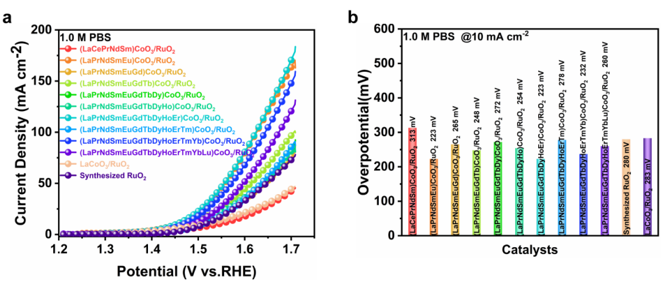


**Figure S27.** (a) OER LSV polarization curves and (b) overpotentials in 1.0 M PBS at a current density of 10 mA cm^-2^ of synthesized RuO_2_, LaCoO_3_/RuO_2_, (LaCePrNdSm)CoO_3_/RuO_2_ (LaPrNdSmEu)CoO_3_/RuO_2_, (LaPrNdSmEuGd)CoO_3_/ RuO_2_, (LaPrNdSmEuGdTb)CoO_3_/RuO_2_, (LaPrNdSmEuGdTbDy)CoO_3_/RuO_2_, (LaPrNdSmEuGdTbDyHo)CoO_3_/RuO_2_, (LaPrNdSmEuGdTbDyHoEr)CoO_3_/RuO_2_, (LaPrNdSmEuGdTbDyHoErTm)CoO_3_/RuO_2_ (LaPrNdSmEuGdTbDyHoErTmYb)CoO_3_/RuO_2_ and (LaPrNdSmEuGdTbDyHoErTmYbLu)CoO_3_/RuO_2_.


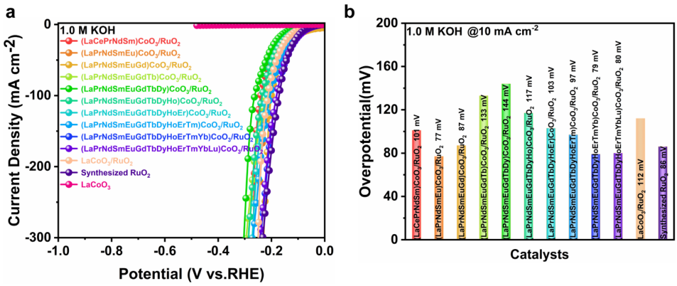


**Figure S28.** (a) HER LSV polarization curves in 1.0 M KOH and (b) overpotentials at a current density of 10 mA cm^-2^ of LaCoO_3_, synthesized RuO_2_, LaCoO_3_/RuO_2_, (LaCePrNdSm)CoO_3_/RuO_2_ (LaPrNdSmEu)CoO_3_/RuO_2_, (LaPrNdSmEuGd)CoO_3_/ RuO_2_, (LaPrNdSmEuGdTb)CoO_3_/RuO_2_, (LaPrNdSmEuGdTbDy)CoO_3_/RuO_2_, (LaPrNdSmEuGdTbDyHo)CoO_3_/RuO_2_, (LaPrNdSmEuGdTbDyHoEr)CoO_3_/RuO_2_, (LaPrNdSmEuGdTbDyHoErTm)CoO_3_/RuO_2_ (LaPrNdSmEuGdTbDyHoErTmYb)CoO_3_/RuO_2_ and (LaPrNdSmEuGdTbDyHoErTmYbLu)CoO_3_/RuO_2_.


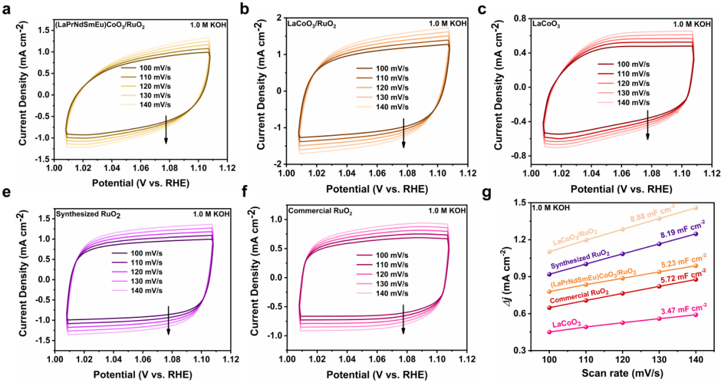


**Figure S29.** (a-e) Cyclic voltammetry curves for (LaPrNdSmEu)CoO_3_/RuO_2_, LaCoO_3_/RuO_2_, LaCoO_3_, synthesized RuO_2_ and commercial RuO_2_ at different scan rates for OER in 1.0 M KOH (100-140 mV s^-1^). (g) Linear plot of capacitive current versus scan rate for these samples.





**Figure S30.** EIS curves for (LaPrNdSmEu)CoO_3_/RuO_2_, LaCoO_3_/RuO_2_, synthesized RuO_2_, commercial RuO_2_ and LaCoO_3_ in 1.0 M KOH.


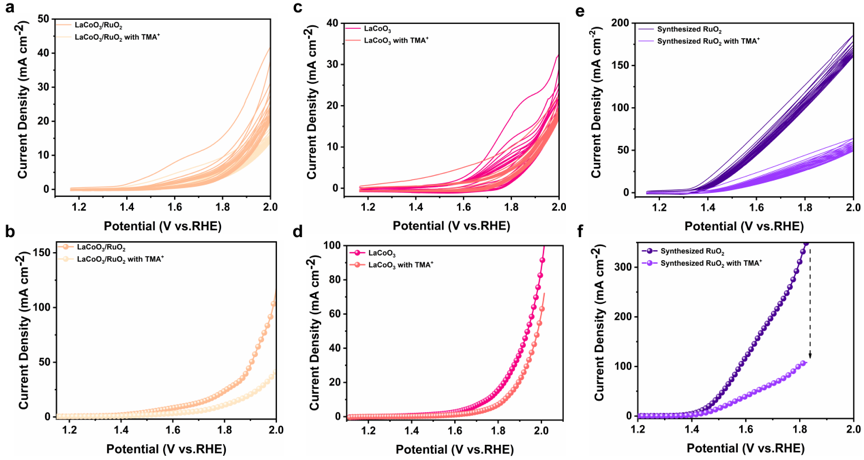


**Figure S31.** (a, c, e) CV and (b, d, f) LSV polarization curves of the OER on LaCoO_3_/RuO_2_, LaCoO_3_ and Synthesized RuO_2_ in 0.5 M H_2_SO_4_ with (5 mL) or without (0 mL) TMA^+^, respectively.


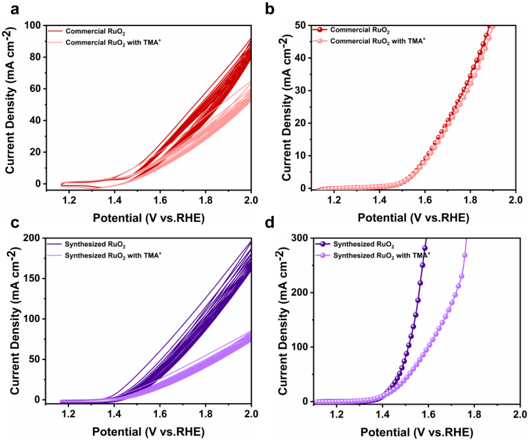


**Figure S32.** (a) CV and (b) LSV polarization curves of the OER on commercial RuO_2_ in 0.5 M H_2_SO_4_ with or without TMA^+^, respectively. (c) CV and (d) LSV polarization curves of the OER on synthesized RuO_2_ in 0.5 M H_2_SO_4_ with or without TMA^+^, respectively.


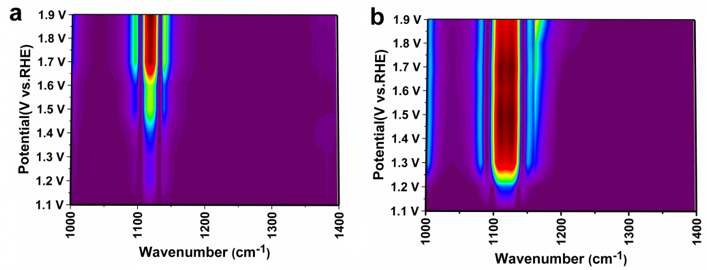


**Figure S33.** The corresponding contour plot of (LaPrNdSmEu)CoO_3_/RuO_2_ in (a) 1 M KOH and (b) 0.5 M H_2_SO_4_ solution.


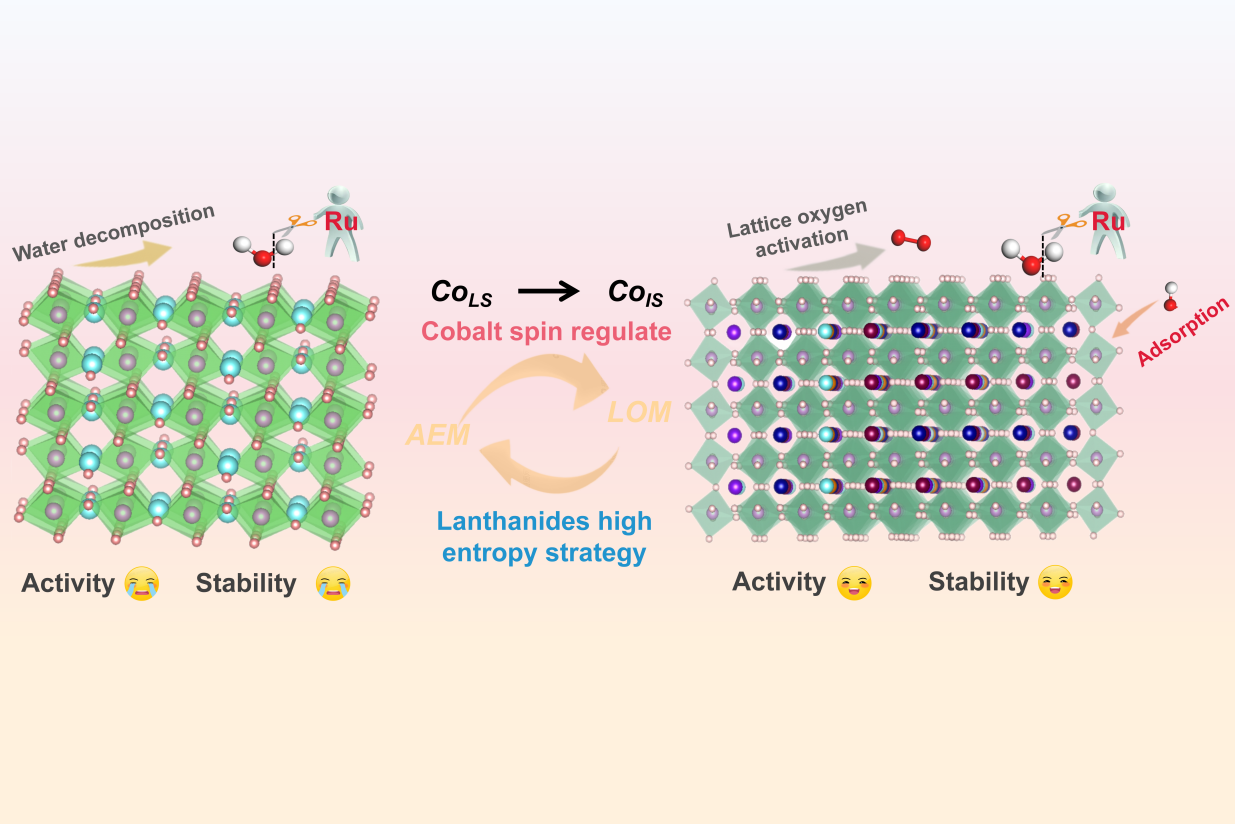


**Figure S34.** Illustration of mechanism shifting and spin regulation of Co in (LaPrNdSmEu)CoO_3_/RuO_2_ controlled by high entropy effects.


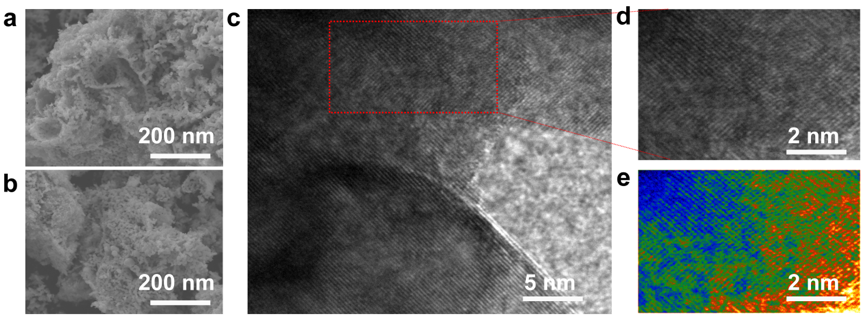


**Figure 35.** (a, b) SEM images and (c-e) HR-TEM images of (LaPrNdSmEu)CoO_3_/RuO_2_ after OER test in 0.5 M H_2_SO_4_ condition.


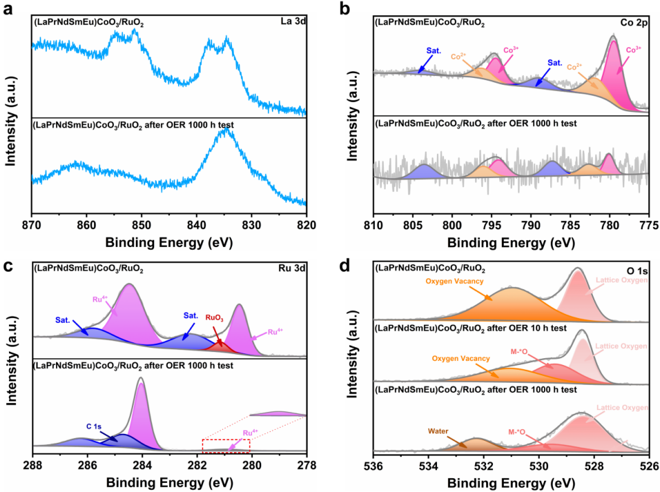


**Figure S36.** (a-c) XPS spectra of La 3d, Ru 3d and Co 2p in (LaPrNdSmEu)CoO_3_/RuO_2_ before and after OER test. (d) O 1s spectrum of (LaPrNdSmEu)CoO_3_/RuO_2_ under different testing times.


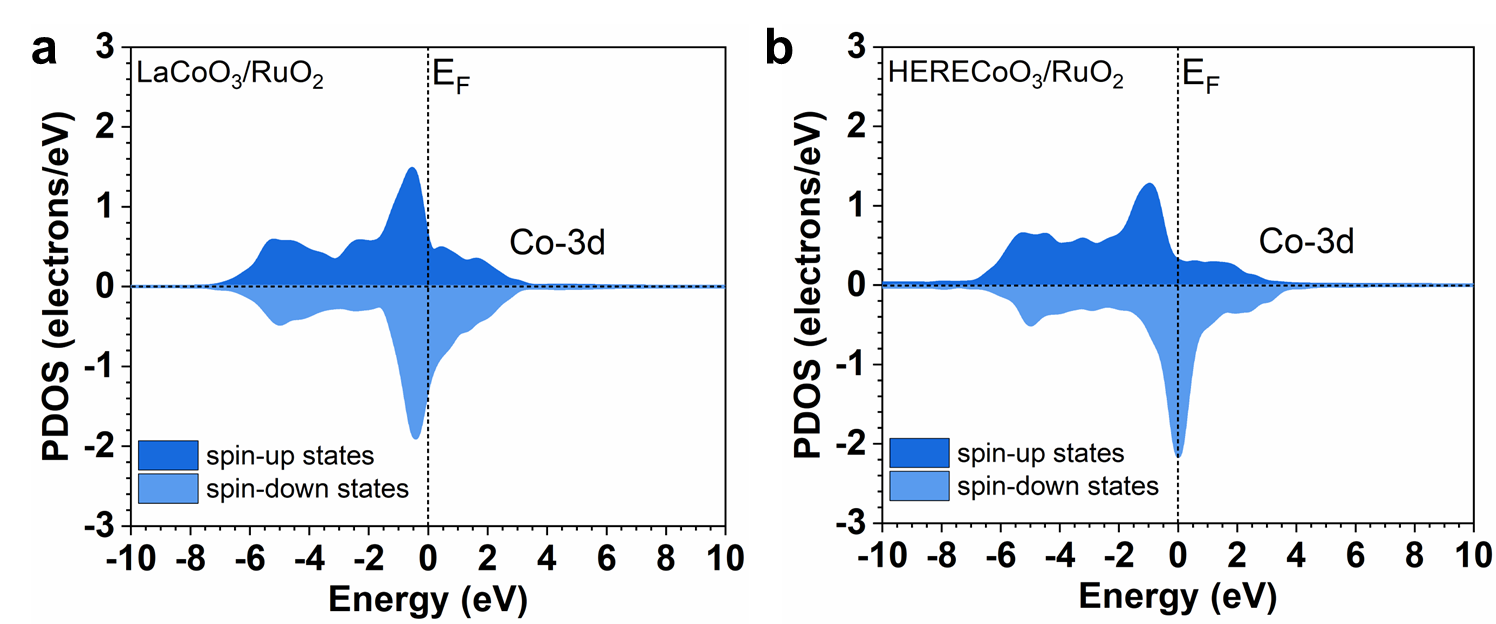


**Figure S37.** The spin polarized PDOS comparison of Co-3d in (a) LaCoO_3_/RuO_2_ and (b) HERECO_3_/RuO_2_.

**Table S1.** The ICP-OES results for Ru content in (HERE)CoO_3_/RuO_2_.

| **Sample** | **Ru content (At %)** |
| --- | --- |
| **(LaCePrNdSm)CoO_3_/RuO_2_** | **2.38 %** |
| **(LaPrNdSmEu)CoO_3_/RuO_2_** | **2.51 %** |
| **(LaPrNdSmEuGd)CoO_3_/RuO_2_** | **2.47 %** |
| **(LaPrNdSmEuGdTb)CoO_3_/RuO_2_** | **2.56 %** |
| **(LaPrNdSmEuGdTbDy)CoO_3_/RuO_2_** | **2.44 %** |
| **(LaPrNdSmEuGdTbDyHo)CoO_3_/RuO_2_** | **2.63 %** |
| **(LaPrNdSmEuGdTbDyHoEr)CoO_3_/RuO_2_** | **2.48 %** |
| **(LaPrNdSmEuGdTbDyHoErTm)CoO_3_/RuO_2_** | **2.57 %** |
| **(LaPrNdSmEuGdTbDyHoErTmYb)CoO_3_/RuO_2_** | **2.43 %** |
| **(LaPrNdSmEuGdTbDyHoErTmYbLu)CoO_3_/RuO_2_** | **2.61 %** |

**Table S2.** EXAFS fitting parameters at the Co K-edge for various samples.

| Sample | Shell | CN^a^ | R(Å)^b^ | σ^2^(Å^2^)^c^ | ΔE_0_(eV)^d^ | K-range/Å^-1^ | R-range/Å | R factor |
| --- | --- | --- | --- | --- | --- | --- | --- | --- |
| Co foil | Co-Co | 12.0* | 2.50±0.01 | 0.0060±0.0003 | 8.4±0.2 | 3.0-12.0 | 1.0-3.0 | 0.0026 |
| CoO | Co-O | 6.0±0.9 | 2.12±0.01 | 0.0107±0.0025 | -1.0±0.7 | 3.2-12.0 | 1.0-3.2 | 0.0059 |
|  | Co-Co | 12.0±1.0 | 3.00±0.01 | 0.0087±0.0008 | -4.7±0.3 |  |  |  |
| Co_3_O_4_ | Co-O | 4.0±0.2 | 1.92±0.01 | 0.0021±0.0006 | -0.1±0.4 | 3.0-12.0 | 1.0-3.5 | 0.0021 |
|  | Co-Co | 3.8±0.4 | 2.85±0.01 | 0.0037±0.0007 | -1.8±0.5 |  |  |  |
|  | Co-Co | 8.2±0.9 | 3.36±0.01 | 0.0070±0.0009 | -2.9±0.4 |  |  |  |
| LaCoO_3_ | Co-O | 5.2±0.3 | 1.90±0.01 | 0.0025±0.0008 | -3.5±0.3 | 2.2-12.0 | 1.1-4.0 | 0.0067 |
|  | Co-La | 7.4±0.8 | 3.32±0.01 | 0.0058±0.0008 | -1.9±0.4 |  |  |  |
|  | Co-Co | 2.7±1.2 | 3.90±0.01 | 0.0015±0.0027 | -9.7±1.3 |  |  |  |
| (LaPrNdSmEu)  CoO_3_ | Co-O | 5.6±0.9 | 1.92±0.01 | 0.0015±0.0025 | -3.1±0.9 | 2.0-9.0 | 1.0-2.1 | 0.0136 |

*^a^CN*, coordination number; *^b^R*, the distance to the neighboring atom; *^c^σ*^2^, Debye-Waller factor , the Mean Square Relative Displacement (MSRD); *^d^ΔE*_0_, inner potential correction; *R* factor indicates the goodness of the fit. *S*0^2^ was fixed to 0.749, accourding to the experimental EXAFS fit of Co foil by fixing *CN* as the known crystallographic value. * This value was fixed during EXAFS fitting, based on the known structure of Co. Error bounds that characterize the structural parameters obtained by EXAFS spectroscopy were estimated as CN ± 20%; R ± 1%; σ2 ± 20%; ΔE0 ± 20%. A reasonable range of EXAFS fitting parameters: 0.700 < *Ѕ*_0_^2^ < 1.000; *CN >* 0; *σ*^2^ > 0 Å^2^; |Δ*E*_0_| < 15 eV; *R* factor < 0.02.

**Table S3.** Performance comparison of (LaPrNdSmEu)CoO_3_/RuO_2_ with the reported Ru-based OER catalysts.

| **Catalyst** | **Electrolytes** | **Overpotential**  **(10 mA cm^-2^)** | **Ref.** |
| --- | --- | --- | --- |
| **(LaPrNdSmEu)CoO_3_/RuO_2_** | **0.5 M H_2_SO_4_** | **115** | **This work** |
| Rh-RuO_2_/G | 0.5 M H_2_SO_4_ | 161 | *Nat Commun.* 2023, *14,* 1412 |
| Ru_1_-Pt_3_Cu | 0.1 M HClO_4_ | 220 | *Nat. Catal.* 2019, *2,* 304-313 |
| Sn_0.1_-RuO_2_@NCP | 0.5 M H_2_SO_4_ | 178 | *Chem. Eng. J.* 2021, *409,* 128155 |
| Ni-Ru@RuO_x_-HL | 0.5 M H_2_SO_4_ | 184 | *Adv. Energy Mater.* 2021, *11,* 2003448 |
| Ru/RuS_2_ | 0.5 M H_2_SO_4_ | 201 | *Angew. Chem. Int. Ed.* 2021, *60,* 12328-12334 |
| Mn-RuO_2_ | 0.5 M H_2_SO_4_ | 158 | *ACS Catal*. 2019, *10,* 1152-1160 |
| CaCuRu_4_O_12_ | 0.5 M H_2_SO_4_ | 171 | *Nat. Commun.* 2019, *10,* 3809 |
| Cr_0.6_Ru_0.4_O_2_ | 0.5 M H_2_SO_4_ | 178 | *Nat. Commun.* 2019, *10,* 162 |
| W_0.2_Er_0.1_Ru_0.7_O_2−δ_ | 0.5 M H_2_SO_4_ | 168 | *Nat. Commun.* 2020, *11,* 5368 |
| UfD-RuO_2_ | 0.5 M H_2_SO_4_ | 179 | *Adv. Energy Mater.* 2023, *9,* 1901313. |
| Ru/S NSs-400 | 0.5 M H_2_SO_4_ | 219 | *Small* 2023, 2208202 |
| NC@Vo-RuO_2_/CNTs | 0.5 M H_2_SO_4_ | 170 | *Adv. Energy Mater.* 2023, 2300152 |
| Nb_0.1_Ru_0.9_O_2_ | 0.5 M H_2_SO_4_ | 204 | *Joule* 2023, *7,* 558-573 |
| Y_2-x_Co_x_Ru_2_O_7−δ_ | 0.5 M H_2_SO_4_ | 275 | *Adv. Funct. Mater.* 2023, *33,* 2208399 |
| Re_0.06_Ru_0.9_4O_2_ | 0.5 M H_2_SO_4_ | 190 | *Nat Commun.* 2023, *14,* 354 |
| Ru@V-RuO_2_/C HMS | 0.5 M H_2_SO_4_ | 176 | *Adv. Mater.* 2023, 2206351 |
| Nd_0.1_RuO_x_ | 0.5 M H_2_SO_4_ | 211 | *Adv. Funct. Mater.* 2023, *33,* 2213304 |
| Ru/Se-RuO_2_ | 0.5 M H_2_SO_4_ | 190 | *Adv. Funct. Mater.* 2023, *33,* 2211102 |
| Ni-RuO_2_ | 0.5 M H_2_SO_4_ | 214 | *Nat. Mater.* 2023, 22, 100-108 |

**Table S4.** Comparison of OER performance of LaPrNdSmEuCoO_3_/RuO_2_ catalyst with recently reported high entropy-based electrocatalysts in alkaline media.

| **Catalyst** | **Overpotential**  **(10 mA cm^-2^)** | **Electrolytes** | **Ref.** |
| --- | --- | --- | --- |
| **LaPrNdSmEuCoO_3_/**  **RuO_2_** | **340 mV** | 1.0 M KOH | ***This work*** |
| FeCoNiCrMo | 260 mV | 1.0 M KOH | *Chem. Eng. J.* 2023, *469,* 144015 |
| K_0.8_Na_0.2_(MgMnFeCoNi)F_3_ | 314 mV | 1.0 M KOH | *J. Am. Chem. Soc.* 2020, *142,* 4550 |
| FeCoNiCuPd | 390 mV | 1.0 M KOH | *Chem. Commun.* 2021, *57,* 2637 |
| La(CrMnFeCo_2_Ni)O_3_ | 325 mV | 1.0 M KOH | *Adv. Funct. Mater.* 2021, *31,* 2101632 |
| CoFeGaNiZn | 370 mV | 1.0 M KOH | *Nano Res.* 2022, *15,* 4799 |
| FeCoNiMnCu | 280 mV | 1.0 M KOH | *Chem. Eng. J.* 2021, *425,* 131533 |
| (Cr_0.2_Mn_0.2_Fe_0.2_Ni_0.2_Zn_0.2_)_3_O_4_ | 295 mV | 1.0 M KOH | *J. Mater. Chem. A* 2022, *10,* 17633 |
| (La_0.6_Sr_0.4_)  (Co_0.2_[FeMnNiMg]_0.8_O_3_) | 320 mV | 1.0 M KOH | *Adv. Funct. Mater.* 2022, *32,* 2112157 |

**Table S5.** Comparison of OER performance of (LaPrNdSmEu)CoO_3_/RuO_2_ catalyst with recently reported advanced electrocatalysts in neutral media.

| **Catalyst** | **Overpotential**  **(10 mA cm^-2^)** | **Electrolytes** | **Ref.** |
| --- | --- | --- | --- |
| **(LaPrNdSmEu)CoO_3_/RuO_2_** | **223 mV** | 1.0 M PBS | ***This work*** |
| ZnFeNiCuCoRu-O | 270 mV | 1.0 M PBS | *Adv. Mater.* 2024, *36,* 2308490 |
| CuCoNiFeMn | 320 mV | 1.0 M PBS | *J. Mater.Chem. A* 2021, *9,* 16841 |
| Ir-CoFe LDH | 323 mV | 1.0 M PBS | *Nano Lett.* 2023, *23,* 5092 |
| CoMoNiS-NF-31 | 405 mV | 1.0 M PBS | *J. Am. Chem. Soc.* 2019, *141,* 10417 |
| RuIr@CoNC | 300 mV | 1.0 M PBS | *ACS Catal.* 2021, *11,* 3402 |
| IrCoOx/C | 470 mV | 1.0 M PBS | *Adv. Mater.* 2018, *30,* 1707522 |
| CoIr-0.2 | 373 mV | 1.0 M PBS | *Adv. Mater.* 2018, *30,* 1707522 |
| CoP@CoOOH | 318 mV | 1.0 M PBS | *Small 2022, 18, 2106012* |
| IrRu@Te | 309 mV | 1.0 M PBS | *ACS Catal.* 2020, *10,* 3571 |

**Table S6.** TOF values of (LaPrNdSmEu)CoO_3_/RuO_2_, LaCoO_3_/RuO_2_, LaCoO_3_, Synthesized RuO_2_ and Commercial RuO_2_ in 0.5 M H_2_SO_4_ solution.

| **Catalysts** | **OER** | |
| --- | --- | --- |
|  | **Overpotential** | **TOF value** |
| **(LaPrNdSmEu)CoO_3_/RuO_2_** | @150 mV | 79.94 s^-1^ |
| **LaCoO_3_/RuO_2_** | @ 300 mV | 3.88 s^-1^ |
| **LaCoO_3_** | @ 300 mV | 1.18 s^-1^ |
| **Synthesized RuO_2_** | @ 300 mV | 32.11 s^-1^ |
| **Commercial RuO_2_** | @ 300 mV | 1.59 s^-1^ |

**Table S7.** TOF values of (HERE)CoO_3_/RuO_2_ in 0.5 M H_2_SO_4_ solution.

| **Sample** | **Overpotential** | **TOF value** |
| --- | --- | --- |
| **(LaCePrNdSm)CoO_3_/RuO_2_** | **@150 mV** | **7.98 s^-1^** |
| **(LaPrNdSmEu)CoO_3_/RuO_2_** | **@150 mV** | **79.94 s^-1^** |
| **(LaPrNdSmEuGd)CoO_3_/RuO_2_** | **@150 mV** | **46.91 s^-1^** |
| **(LaPrNdSmEuGdTb)CoO_3_/RuO_2_** | **@150 mV** | **26.53 s^-1^** |
| **(LaPrNdSmEuGdTbDy)CoO_3_/RuO_2_** | **@150 mV** | **18.10 s^-1^** |
| **(LaPrNdSmEuGdTbDyHo)CoO_3_/RuO_2_** | **@150 mV** | **15.72 s^-1^** |
| **(LaPrNdSmEuGdTbDyHoEr)CoO_3_/RuO_2_** | **@150 mV** | **10.32 s^-1^** |
| **(LaPrNdSmEuGdTbDyHoErTm)CoO_3_/RuO_2_** | **@150 mV** | **5.16 s^-1^** |
| **(LaPrNdSmEuGdTbDyHoErTmYb)CoO_3_/RuO_2_** | **@150 mV** | **7.58 s^-1^** |
| **(LaPrNdSmEuGdTbDyHoErTmYbLu)CoO_3_/RuO_2_** | **@150 mV** | **3.58 s^-1^** |

**Table S8.** The content of Ru and Co ions dissolved in the electrolyte after OER test in 0.5 M H_2_SO_4_ solution from ICP-OES results.

| **Catalyst** | **Pristine Ru content (mg/L)** | **Ru content (mg/L) after OER test** | **Ru Loss (mg/L)** |
| --- | --- | --- | --- |
| **LaCoO_3_/RuO_2_** | 0.48 | 0.19 | 0.29 |
| **(LaPrNdSmEu)CoO_3_/RuO_2_** | 0.46 | 0.36 | 0.10 |
| **(LaPrNdSmEuGdTbDyHoErTmYbLu)CoO_3_/RuO_2_** | 0.48 | 0.40 | 0.08 |
| **Catalyst** | **Pristine Co content (mg/L)** | **Co content (mg/L) after OER test** | **Co Loss (mg/L)** |
| **LaCoO_3_/RuO_2_** | 1.43 | 0.71 | 0.72 |
| **(LaPrNdSmEu)CoO_3_/RuO_2_** | 1.43 | 1.26 | 0.17 |
| **(LaPrNdSmEuGdTbDyHoErTmYbLu)CoO_3_/RuO_2_** | 1.44 | 1.34 | 0.10 |

**Table S9** The content of RE ions dissolved in the electrolyte after OER test in 0.5 M H_2_SO_4_ solution from ICP-OES results.

| **(LaPrNdSmEu)CoO_3_/RuO_2_** | **Pristine**  **content (mg/L)** | **content (mg/L) after OER test** | **Loss (mg/L)** |
| --- | --- | --- | --- |
| La | 0.502 | 0.445 | 0.055 |
| Pr | 0.501 | 0.46 | 0.043 |
| Nd | 0.504 | 0.45 | 0.051 |
| Sm | 0.503 | 0.46 | 0.044 |
| Eu | 0.502 | 0.445 | 0.055 |
| **Total** | **2.512** | **2.264** | **0.248** |
| **(LaPrNdSmEuGdTbDyHoErTmYbLu)CoO_3_/RuO_2_** | **Pristine**  **content (mg/L)** | **content (mg/L) after OER test** | **Loss (mg/L)** |
| La | 0.192 | 0.179 | 0.013 |
| Pr | 0.191 | 0.179 | 0.012 |
| Nd | 0.192 | 0.180 | 0.012 |
| Sm | 0.193 | 0.181 | 0.012 |
| Eu | 0.192 | 0.179 | 0.013 |
| Gd | 0.192 | 0.180 | 0.012 |
| Tb | 0.194 | 0.180 | 0.014 |
| Dy | 0.194 | 0.181 | 0.013 |
| Ho | 0.193 | 0.182 | 0.011 |
| Er | 0.194 | 0.182 | 0.012 |
| Tm | 0.195 | 0.181 | 0.014 |
| Yb | 0.194 | 0.181 | 0.013 |
| Lu | 0.194 | 0.182 | 0.012 |
| **Total** | **2.510** | **2.347** | **0.163** |

**Reference**

[1] S.-J. Clark, M.-D. Segall, C.-J. Pickard, P.-J. Hasnip, M.-J. Probert, K. Refson, M.-C. Payne, First principles methods using CASTEP, Z. Kristallogr., 2005, 220, 567-570.

[2] J.-P. Perdew, K. Burke, M. Ernzerhof, Generalized gradient approximation made simple, Phys. Rev. Lett., 1996, 77, 3865-3868.

[3] P.-J. Hasnip, C.-J. Pickard, Electronic energy minimisation with ultrasoft pseudopotentials.Comput. Phys. Commun., 2006, 174, 24-29.

[4] J.-P. Perdew, J.-A. Chevary, S.-H. Vosko, K.-A. Jackson, M.-R. Pederson, D.-J. Singh, C. Fiolhais, Atoms, molecules, solids, and surfaces: applications of the generalized gradient approximation for exchange and correlation, Phys. Rev. B, 1992, 46, 6671-6687.

[5] J.-D. Head, M.-C. Zerner, A broyden-fletcher-goldfarb-shanno optimization procedure for molecular geometries, Chem. Phys. Lett., 1985, 122, 264-270.
